# Reproductive Toxicity Induced by Serotonin‐Norepinephrine Reuptake Inhibitors: A Pharmacovigilance Analysis From 2004 to 2023 Based on the FAERS Database

**DOI:** 10.1111/cns.70176

**Published:** 2024-12-13

**Authors:** Yujia Xi, Zhuocheng Bao, Qiang Guo, Jingqi Wang, Zhinan Jing, Jingkai Di, Ke Yang

**Affiliations:** ^1^ Department of Urology Second Hospital of Shanxi Medical University Taiyuan China; ^2^ Male Reproductive Medicine Center Shanxi Medical University Jinzhong China; ^3^ Department of Orthopedics Second Hospital of Shanxi Medical University Taiyuan China

**Keywords:** disproportionality analysis, drug safety, FAERS, pharmacovigilance, reproductive toxicity, serotonin‐norepinephrine reuptake inhibitors

## Abstract

**Aim:**

Serotonin‐norepinephrine reuptake inhibitors (SNRIs) have been extensively utilized for the treatment of depression and anxiety disorders. Clinical trials and real‐world data suggest that SNRIs may cause reproductive toxicity. To comprehensively assess this association, we conducted a pharmacovigilance study.

**Methods:**

We utilized various disproportionality analysis algorithms, including reporting odds ratio (ROR), proportional reporting ratio (PRR), bayesian confidence propagation neural network (BCPNN), and multi‐item gamma poisson shrinker (MGPS), to assess the significance of reproductive toxicity‐related adverse events (AEs) reported to FDA Adverse Event Reporting System (FAERS) from January 2004 to December 2023, with subgroup analysis conducted by sex and age.

**Results:**

Duloxetine and venlafaxine were associated with 14 and 25 AE signals related to reproductive toxicity, respectively, with erectile dysfunction (ED) and retrograde ejaculation identified as shared important medical events (IMEs). ED had the highest reporting frequency, strongest in venlafaxine‐treated patients under 45 years (ROR 4.34, PRR 4.33, IC 2.09, EBGM 4.25). Retrograde ejaculation was newly identified. With decreasing incidence, venlafaxine's median ED onset was 122.5 days and duloxetine's 38 days.

**Conclusion:**

Our study provides evidence through an extensive analysis of the large‐scale real‐world FAERS database, aiding healthcare professionals in mitigating, and prioritizing SNRI‐related reproductive toxicity AEs.

## Graphical abstract

A comprehensive analysis of the large‐scale real‐world FAERS database was conducted to evaluate the relationship between SNRIs and adverse reproductive system events. In patients treated with SNRIs, males should focus on erectile dysfunction and retrograde ejaculation, while females should be alert to potential associations with ovarian cysts.

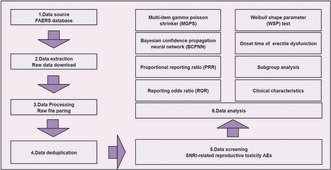

## 1 Introduction

Serotonin‐norepinephrine reuptake inhibitors (SNRIs), commonly referred to as dual‐action antidepressants, work by inhibiting presynaptic transporter proteins that reabsorb serotonin and norepinephrine [1]. This inhibition prevents the reuptake of these neurotransmitters, thereby prolonging their presence in the synaptic cleft [2]. As a result, serotonin and norepinephrine can bind to receptors on neurons for longer, potentiating their effects. This enhancement helps to regulate mood, emotions, and cognitive function, thereby alleviating symptoms of mood disorders such as depression and anxiety [3]. Medication is currently the main treatment for depression, with SNRIs widely endorsed by authoritative guidelines as a first‐line therapy [4]. Besides depression, SNRIs are also used to treat other conditions, including anxiety disorders and chronic pain, especially neuropathic pain [5, 6].

In the past decade, concerns have grown about the interference of xenobiotics, such as medications, as well as occupational and environmental exposures, with human reproduction, leading to reproductive toxicity [7, 8, 9]. Therefore, the reproductive toxicity of SNRIs has drawn the attention of clinicians and researchers. The underlying mechanisms of reproductive toxicity are intricate and not well comprehended [10, 11]. Serotonin and noradrenaline exert effects on both the brain and the genitalia. Among these, serotonin is the primary neurotransmitter that negatively affects sex drive and sexual function, both centrally and peripherally [12]. Specifically, the stimulation of serotonin (5‐HT) 2A receptors in the central serotonergic system is believed to play a major role in the development of sexual dysfunction caused by antidepressant medication [10].

The representative drugs of the commonly used first‐line SNRIs are venlafaxine and duloxetine. According to Rothmore's research [13], SNRIs pose a high risk of sexual dysfunction. Still, there is an inadequate amount of evidence to make a comparison of the individual risks across SNRIs and selective serotonin reuptake inhibitors (SSRIs), as well as among different SNRIs. Additionally, compared to venlafaxine, duloxetine carries a lower risk, although additional data is required to substantiate this assertion. A recent systematic review demonstrated a significantly increased incidence of erectile dysfunction (ED) associated with the use of SNRIs (duloxetine, venlafaxine) compared to placebo, while the incidence associated with SSRIs showed no significant increase [14]. Therefore, the safety concerns regarding the reproductive system in the treatment of depression with SNRIs remain a topic of ongoing debate. It is crucial to emphasize that a systematic review mostly relies on data sourced from published articles, which may not always offer an adequate dataset. On the other hand, the FDA Adverse Event Reporting System (FAERS), which is a publicly accessible database in the United States, contains numerous adverse event (AE) reports voluntarily submitted by consumers, manufacturers, healthcare professionals, and other stakeholders. Its primary goal is to aid the FDA in post‐marketing safety monitoring of biological and pharmaceutical products. Therefore, employing adverse reaction database mining using data from the FAERS database is a reliable method to establish the association between drugs and AEs. FAERS's comprehensive and constantly updated library allows for more precise analysis of adverse reaction data, enabling a more accurate understanding of real‐world study dynamics. Utilizing data mining techniques in a vast database of spontaneous AE reports is becoming essential for conducting pharmaceutical safety studies in pharmacovigilance research [15, 16]. Furthermore, there is currently no existing data mining research on AEs associated with SNRIs using the FAERS database.

The objective of our study is to analyze and assess the possible correlation between SNRIs and reproductive toxicity using FAERS data, aiming to raise awareness, enhance the management, and promote additional research to provide clinical guidance.

## 2 Methods

### 2.1 Data Sources

We conducted a retrospective analysis of pharmacovigilance utilizing the FAERS database, an internationally acknowledged and openly available source for AE reports. The FAERS database is updated quarterly, and users have free access to the complete dataset at https://fis.fda.gov/extensions/FPD‐QDE‐FAERS/FPD‐QDE‐FAERS.html. Data is available for download in both XML and ASCII formats directly from the FDA website. The FAERS quarterly file package consists of seven data files: DEMO (patient demographic and administrative information), DRUG (drug information), REAC (coded for the AEs), OUTC (patient outcomes), RPSR (report sources), THER (therapy start and end dates for reported drugs), INDI (indications for drug administration), and information on deleted cases. The files provide comprehensive information regarding AEs, which are connected to the PRIMARYID, CASEID, and drug_seq.

For our investigation, we analyzed reports uploaded to the FAERS database between January 1, 2004, and December 31, 2023. Due to the nature of data updating, it is unavoidable that there will be duplicate reporting in FAERS. Hence, we eradicated redundant entries according to the particular criteria outlined by the FDA. If the CASEIDs were identical, we chose the more recent FDA_DT. When both the CASEIDs and FDA_DTs were the same, we chose the PRIMARYID with a greater numerical value [17]. Since the study was carried out using de‐identified publically available data, ethical approval was not required.

### 2.2 Procedures

The Medical Dictionary for Regulatory Activities (MedDRA) terminology, based on preferred terms (PTs) for signs and symptoms, is employed for the classification of adverse reactions in the FAERS database, where drugs are classified into four patterns: PS (primary suspect), SS (second suspect), C (concomitant), and I (interacting) [18, 19]. To improve the accuracy of our research, we limited our examination to DRUG files where the drug's role_cod was designated as “PS.” Two SNRIs were chosen using the MeSH Browser, with their generic and brand names identified. Information regarding the clinical features, including gender, age, reporting area, reporter, duration of treatment, date of AE, and year of report, was collected for reports that associated reproductive toxicity with SNRIs.

The interval between AE occurrence (EVENT_DT, DEMO file) and SNRI use (START_DT, THER file) is used to estimate time‐to‐onset (TTO) [20]. While integrating the median, quartiles, minimum, maximum, and Weibull shape parameter (WSP) evaluation for TTO, data issues such EVENT_DT preceding START_DT, erroneous dates, and nonexistent data are excluded to assure study precision. The WSP analysis evaluates AE occurrence over time using the scale (*α*) and shape (*β*) parameters, which determine the distribution function's amplitude and structure. A *β* value of < 1 (95% CI < 1) suggests decreasing risk over time (early failure type), *β* equal to or proximate to 1 (95% CI includes 1) indicates constant risk (random failure type), and *β* > 1 (95% CI does not include 1) indicates increasing risk (wear‐out failure type) [21]. Kaplan–Meier plots show cumulative AE incidence, and log‐rank tests compare groups. We consider statistical significance as *p* < 0.05.

### 2.3 Data Analysis

The disproportionality analysis algorithm is designed as a data mining technique to identify signals of AEs in the spontaneous reporting system. Its primary goal is to identify drug‐AE associations that are statistically significant and to estimate the effect size of these associations. Within the framework of disproportionality analysis, it is postulated that there is no inherent correlation between the medicine and the adverse reaction. The study aims to detect any inconsistencies by comparing the observed frequency of drug‐adverse reaction pairs with the anticipated frequency of reports. The predicted number of reports corresponds to the average frequency of reporting when there is no association between the medicine and the adverse reaction, serving as a reference point for assessing false‐positive reports (Table S1).

However, it is important to choose disproportionality analysis techniques judiciously, as different approaches will yield varied outcomes. The signals produced by various methodologies can also complement one another, providing significant benefits to pharmaceutical research. In disproportionality analysis, a modified relative risk (RR) is the primary statistical measure used to assess the association between the drug and adverse reaction pairs. To ensure the robustness of our results, we utilized multiple disproportionality analysis methods, including two non‐Bayesian approaches—reporting odds ratio (ROR) and proportional reporting ratio (PRR)—as well as two Bayesian methods—bayesian confidence propagation neural network (BCPNN) and multi‐item gamma Poisson shrinker (MGPS) [22]. Non‐Bayesian methods like ROR are effective for early signal detection, whereas Bayesian techniques are better at identifying unique signals, especially with limited reports [23]. A higher disproportionality score indicates a greater likelihood of a drug causing a specific AE. A signal is considered present if the lower 95% CI of the ROR exceeds 1 and there are at least three reports of the suspected drug and adverse reaction [24]. The formulas and criteria for these methods are detailed in Table S2. Additionally, we performed subgroup analyses to explore the relationship between SNRIs and adverse effects by age (< 45 [child and youth], 45–59 [middle age], > 59 [elder]) and gender (male and female).

### 2.4 Statistical Analysis

The Kolmogorov–Smirnov test assessed normality. Data are presented as mean ± standard deviation for normal distributions and median (IQR) for non‐normal distributions. We report categorical variables as frequencies and percentages, compare them using Pearson's chi‐squared (*χ* ^2^) or Fisher's exact test, and adjust *p*‐values for false discovery rate (FDR). Two‐tailed *p* < 0.05 indicated statistical significance. Data analysis and visualization were done in Microsoft Office 2019 or R 4.3.3 (https://posit.co/download/rstudio‐desktop/).

## 3 Results

### 3.1 Descriptive Analysis

**FIGURE 1:**
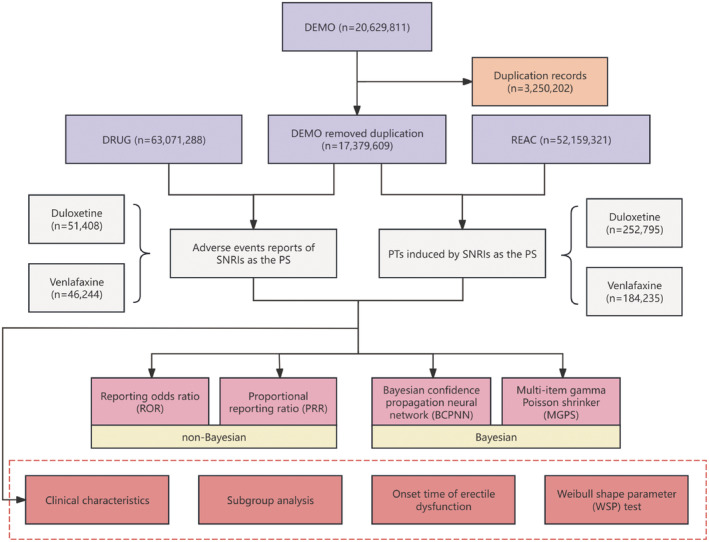
The flow diagram of selecting SNRl‐related AEs from FAERS database.

Figure 1 depicts the flowchart of the multi‐step procedure used in this study for data extraction, processing, and analysis. Between January 2004 and December 2023, the FAERS database recorded a total of 20,629,811 case reports. After removing duplicates, 17,379,609 unique patient cases remained, with 52,159,321 reported AEs. Of these, 51,408 AEs were associated with duloxetine and 46,244 with venlafaxine. The clinical characteristics of these drugs are detailed in Table 1. Among the reports of AEs for duloxetine and venlafaxine, 48,357 and 41,570 cases, respectively, included the submitter's gender. In these cases, 69.4% of duloxetine reports and 64.2% of venlafaxine reports were from females. Age distribution for duloxetine AEs was 18.9% for those under 45, 19.9% for ages 45–59, and 19.8% for those over 59. For venlafaxine, the rates were 24.8% (< 45), 21.1% (45–59), and 20.0% (> 59). In reports with patient weight data, the 50–100 kg group was the largest for both medications, with 16.5% for duloxetine and 26.0% for venlafaxine. Reports were primarily submitted by consumers (57.3% for duloxetine and 41.2% for venlafaxine) and healthcare professionals (24.5% for duloxetine and 24.2% for venlafaxine), which enhances the reliability of the AE information. Among serious outcomes, 32.6% of duloxetine‐related and 36.3% of venlafaxine‐related reports were classified as “other serious outcomes.” Hospitalization was the most common serious outcome (15.5% for duloxetine and 19.4% for venlafaxine), followed by death rates (3.2% and 6.5%) and life‐threatening events (2.9% and 4.3%).

**TABLE 1:** Clinical characteristics of reports with SNRls from the FAERS database.

Characteristics	Duloxetine (*N*, %)	Venlafaxine (*N*, %)
Total number of reports	51,408	46,244
Gender		
Female	35,666 (69.4%)	29,686 (64.2%)
Male	12,691 (24.7%)	11,884 (25.7%)
Unknown	3051 (5.9%)	4674 (10.1%)
Age (year)		
< 45	9698 (18.9%)	11,448 (24.8%)
45–59	10,245 (19.9%)	9736 (21.1%)
> 59	10,172 (19.8%)	9246 (20.0%)
Unknown	21,293 (41.4%)	15,814 (34.2%)
Weight		
< 50 kg	702 (1.4%)	1657 (3.6%)
50–100	8488 (16.5%)	12,034 (26.0%)
> 100 kg	1604 (3.1%)	1811 (3.9%)
Unknown	40,614 (79.0%)	30,742 (66.5%)
Reported person		
Consumer (CN)	29,433 (57.3%)	19,037 (41.2%)
Health professional (HP)	1109 (2.2%)	2625 (5.7%)
Lawyer (LW)	727 (1.4%)	208 (0.4%)
Physician (MD)	12,606 (24.5%)	11,206 (24.2%)
Other health‐professional (OT)	2622 (5.1%)	6693 (14.5%)
Pharmacist (PH)	2105 (4.1%)	3241 (7.0%)
Registered Nurse (RN)	29 (0.1%)	18 (0.0%)
Unknown	2777 (5.4%)	3216 (7.0%)
Serious outcome		
Congenital anomaly (CA)	230 (0.4%)	1027 (1.8%)
Death (DE)	1836 (3.2%)	3630 (6.5%)
Disability (DS)	1430 (2.5%)	2027 (3.6%)
Hospitalization (HO)	8974 (15.5%)	10,811 (19.4%)
Life‐threatening (LT)	1699 (2.9%)	2395 (4.3%)
Other serious outcomes (OT)	18,908 (32.6%)	20,152 (36.3%)
Required intervention (RI)	296 (0.5%)	282 (0.5%)
Unknown	24,592 (42.4%)	15,264 (27.5%)

Abbreviation: *N*, number of adverse event reported.

**FIGURE 2:**
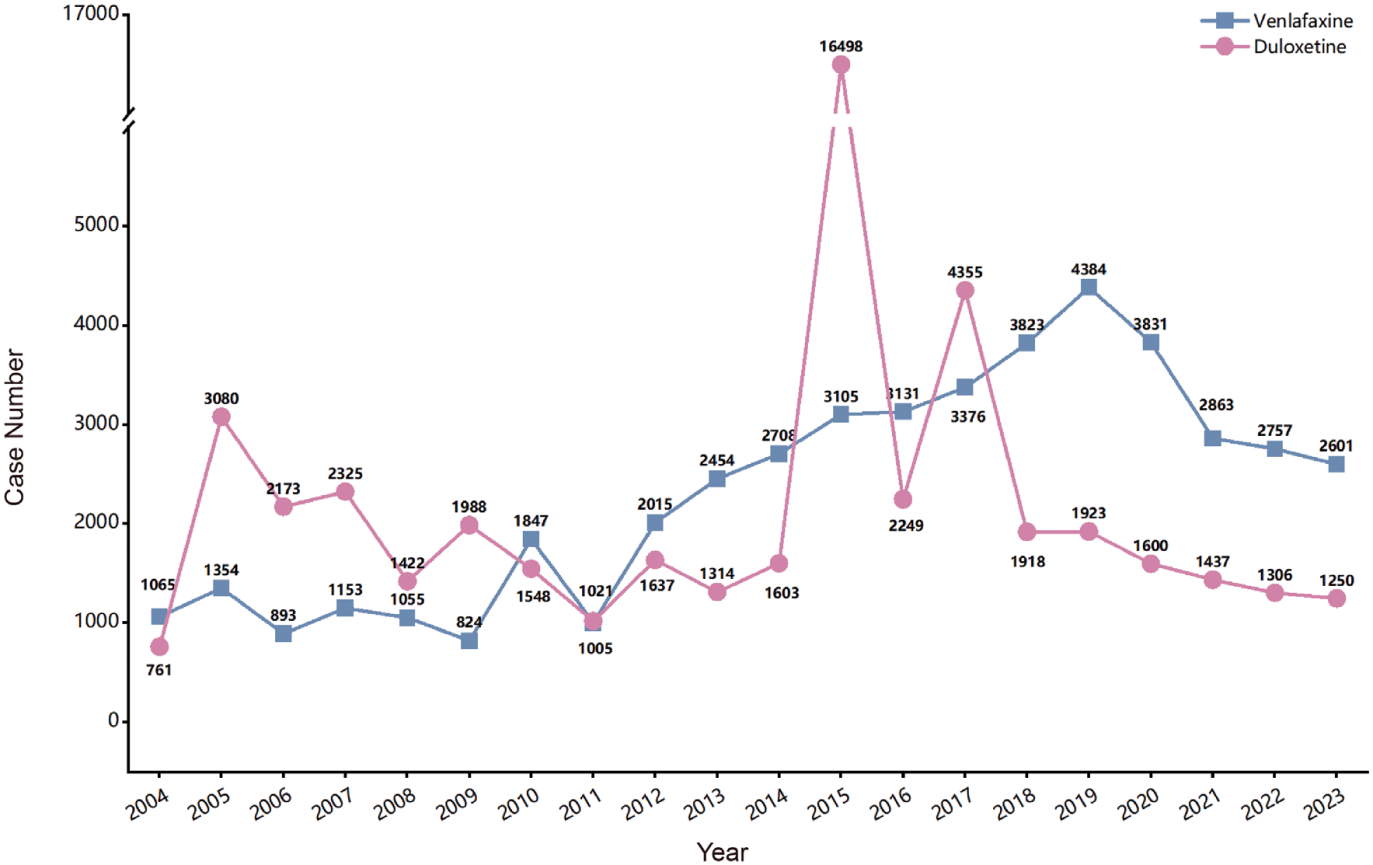
The annual distribution of AE reports related to duloxetine (A) and venlafaxine (B) from 2004 to 2023.

The top five countries reporting duloxetine‐related AEs were the United States (72.3%), Great Britain (2.6%), Japan (2.5%), Canada (2.1%), and France (1.9%). For venlafaxine, the top reporting countries were the United States (55.1%), France (6.5%), Great Britain (5.8%), Germany (5.6%), and Canada (2.9%) (Table 2). The yearly distribution of AE reports related to venlafaxine and duloxetine is shown in Figure 2. Duloxetine‐related reports peaked in 2015 with 16,498 reports, and the lowest number was in 2004 with 761 reports. For venlafaxine, the highest number of reports was in 2019 with 4384, while the lowest was in 2009 with 824 reports.

**TABLE 2:** The top five countries reporting the highest number of SNRI‐related AEs.

Reported countries (top five)	*N*, %
Duloxetine	
United States	37,193 (72.3%)
Great Britain	1320 (2.6%)
Japan	1310 (2.5%)
Canada	1076 (2.1%)
France	980 (1.9%)
Venlafaxine	
United States	25,502 (55.1%)
France	3001 (6.5%)
Great Britain	2666 (5.8%)
Germany	2605 (5.6%)
Canada	1323 (2.9%)

Abbreviation: *N*, number of adverse event reported.

### 3.2 Disproportionality Analysis

**FIGURE 3:**
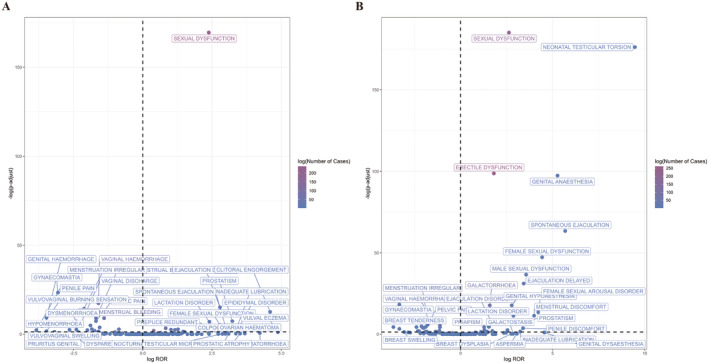
Reporting odds ratios (ROR) for all PTs within the SOC of reproductive system and breast disorders, along with a volcano plot for duloxetine (A) and venlafaxine (B). The horizontal axis shows the log_2_ROR, while the vertical axis displays the negative log of FDR‐corrected p‐values from the chi‐squared (*χ* ^2^) test. Dot color indicates the exponential case report count, with deeper purple reflecting more reports.

Using the ROR method, we generated a volcano chart of 163 duloxetine and 137 venlafaxine PTs for reproductive and breast diseases (Figure 3). The *x*‐axis shows log_2_ROR, while the *y*‐axis presents FDR‐corrected *p*‐values' negative logarithm. We clarified that positive values on the *x*‐axis indicate a stronger reporting association with a specific AE, while positive values on the *y*‐axis denote statistical significance. Dark purple indicates more case reports. AEs in the upper‐right corner had higher signal intensity and statistical significance. Full results are available in Tables S3 and S4. Positive signals were those with at least three reports and a lower 95% CI for the ROR > 1, excluding breast‐related AEs. Duloxetine had 14 reproductive toxicity signals and venlafaxine has 25. In Table 3, AE reports and signal strengths are listed. These signals focus on SNRIs' effects on sexual function and reproductive organ health. Three PTs match important medical events (IMEs): ED, retrograde ejaculation, and hemorrhagic ovarian cysts.

**TABLE 3:** Signal detection of SNRI‐related reproductive toxicity AEs.

Drug	Preferred terms	*N*	ROR (95% two‐sided CI)	PRR (95% two‐sided CI)	*χ* ^2^	IC(IC025)	EBGM (EBGM05)
Duloxetine	Sexual dysfunction^a^	237	5.22 (4.59–5.94)	5.22 (5.09–5.34)	787.59	2.35 (0.69)	5.11 (4.59)
	**Erectile dysfunction**	135	1.31 (1.1–1.55)	1.31 (1.14–1.48)	9.64	0.38 (−1.28)	1.3 (1.13)
	Ejaculation disorded	26	2.02 (1.37–2.97)	2.02 (1.64–2.41)	13.29	1.01 (−0.66)	2.01 (1.46)
	Priapism	23	1.56 (1.03–2.35)	1.56 (1.15–1.97)	4.56	0.64 (−1.03)	1.55 (1.1)
	Ejaculation failure	19	2.23 (1.42–3.5)	2.23 (1.78–2.68)	12.74	1.15 (−0.52)	2.22 (1.52)
	Ejaculation delayed^a^	16	6.9 (4.19–11.36)	6.9 (6.4–7.4)	78.12	2.75 (1.08)	6.71 (4.42)
	**Retrograde ejaculation**	8	2.75 (1.37–5.52)	2.75 (2.05–3.44)	8.77	1.45 (−0.22)	2.72 (1.52)
	**Haemorrhagic ovarian cyst**	7	2.84 (1.35–5.99)	2.84 (2.09–3.59)	8.23	1.49 (−0.18)	2.82 (1.51)
	Female sexual dysfunction^a^	6	6.96 (3.09–15.7)	6.96 (6.15–7.77)	29.62	2.76 (1.08)	6.76 (3.42)
	Prostatism^a^	6	9.4 (4.15–21.32)	9.4 (8.59–10.22)	43.09	3.18 (1.49)	9.04 (4.56)
	Pelvic haematoma	5	2.72 (1.13–6.58)	2.72 (1.84–3.61)	5.38	1.43 (−0.24)	2.7 (1.29)
	Penile discharge^a^	4	3.37 (1.25–9.04)	3.37 (2.38–4.35)	6.55	1.73 (0.06)	3.33 (1.46)
	Clitoral engorgement^a^	4	24.16 (8.57–68.08)	24.16 (23.12–25.19)	79.44	4.44 (2.71)	21.72 (9.13)
	Inadequate lubrication^a^	3	10.44 (3.27–33.31)	10.44 (9.28–11.6)	24.37	3.32 (1.61)	9.98 (3.78)
Venlafaxine	Erectile dysfunction^a^	261	3.49 (3.09–3.95)	3.49 (3.37–3.61)	458.39	1.79 (0.12)	3.46 (3.12)
	Sexual dysfunction^a^	204	6.15 (5.35–7.07)	6.15 (6.01–6.28)	860.39	2.59 (0.93)	6.04 (5.37)
	Ejaculation disorded	29	3.1 (2.15–4.47)	3.1 (2.74–3.47)	40.84	1.62 (−0.04)	3.08 (2.27)
	Priapism	26	2.42 (1.65–3.56)	2.42 (2.04–2.81)	21.51	1.27 (−0.4)	2.41 (1.74)
	Uterine disorded	21	2.86 (1.86–4.4)	2.86 (2.43–3.29)	25.14	1.51 (−0.16)	2.84 (1.98)
	Male sexual dysfunction^a^	19	11.73 (7.41–18.56)	11.73 (11.27–12.19)	179.03	3.5 (1.83)	11.3 (7.7)
	Ejaculation failure	18	2.9 (1.82–4.61)	2.9 (2.44–3.36)	22.18	1.53 (−0.14)	2.88 (1.95)
	Ejaculation delayed^a^	18	10.71 (6.69–17.15)	10.71 (10.24–11.18)	152.72	3.37 (1.7)	10.36 (6.99)
	Genital hypoaesthesia^a^	18	6.84 (4.28–10.91)	6.83 (6.37–7.3)	87.54	2.74 (1.07)	6.7 (4.53)
	Vulvovaginal dryness	17	1.75 (1.09–2.83)	1.75 (1.28–2.23)	5.49	0.81 (−0.86)	1.75 (1.17)
	Menopausal symptoms	16	2.44 (1.49–3.99)	2.44 (1.95–2.93)	13.5	1.28 (−0.39)	2.43 (1.61)
	Genital anesthesia^a^	15	38.47 (22.43–65.99)	38.47 (37.93–39.01)	481.75	5.09 (3.4)	33.97 (21.63)
	Female sexual dysfunction^a^	13	21.57 (12.27–37.92)	21.57 (21.01–22.14)	236.94	4.33 (2.65)	20.11 (12.55)
	Penile size reduced	8	2.71 (1.35–5.44)	2.71 (2.01–3.41)	8.55	1.43 (−0.24)	2.69 (1.5)
	Spontaneous ejaculation^a^	8	51.3 (24.15–108.96)	51.29 (50.54–52.05)	333.81	5.44 (3.73)	43.56 (23.19)
	**Retrograde ejaculation** ^a^	7	3.3 (1.56–6.95)	3.3 (2.55–4.04)	11.07	1.71 (0.04)	3.27 (1.75)
	Testicular atrophy	6	2.35 (1.05–5.25)	2.35 (1.55–3.15)	4.62	1.23 (−0.44)	2.34 (1.19)
	Haematospermia	6	2.77 (1.24–6.19)	2.77 (1.97–3.57)	6.72	1.46 (−0.21)	2.75 (1.4)
	Female sexual arousal disorded^a^	5	18.56 (7.51–45.88)	18.56 (17.66–19.46)	77.94	4.13 (2.43)	17.48 (8.2)
	Neonatal testicular torsion^a^	5	705.3 (136.83–3635.44)	705.28 (703.64–706.92)	1004.69	7.66 (5.71)	202.22 (51.28)
	Prostatism^a^	5	10.69 (4.38–26.1)	10.68 (9.79–11.58)	42.3	3.37 (1.69)	10.33 (4.89)
	Menstrual discomfort^a^	5	15.85 (6.44–39.02)	15.85 (14.95–16.75)	65.86	3.91 (2.22)	15.06 (7.09)
	Painful ejaculation^a^	4	5.82 (2.16–15.66)	5.82 (4.83–6.81)	15.63	2.52 (0.84)	5.72 (2.5)
	Genital paraesthesia^a^	3	3.6 (1.15–11.25)	3.6 (2.46–4.74)	5.57	1.84 (0.16)	3.57 (1.38)
	Penile discomfort^a^	3	10.45 (3.3–33.08)	10.45 (9.3–11.6)	24.72	3.34 (1.64)	10.11 (3.86)

*Note:* Text in bold signifies that the signal is categorized as an important medical events (IMEs). IMEs are developed and updated by European Medicines Agency (EMA).

Abbreviations: CI, confidence interval; EBGM, empirical Bayesian geometric mean; IC, information component; IC025 and EBGM05, lower one‐sided for IC and EBGM, respectively; *N*, number of adverse event reported; PRR, proportional reporting ratio; ROR, reporting odds ratio; *χ* ^2^, chi‐squared.

^a^ Adhering to the four algorithms.

Subgroup studies by gender and age showed different AE patterns (Tables 4 and 5, Tables S5 and S6). In male patients, both ED and retrograde ejaculation were observed across most age subgroups. ED had the highest reporting frequency, particularly notable in patients under 45 years receiving venlafaxine (ROR 4.34, PRR 4.33, IC 2.09, EBGM 4.25). In female patients, therapy with duloxetine caused hemorrhagic ovarian cyst. Furthermore, within the 45–59 age subgroup of patients undergoing venlafaxine treatment, a novel IME was recognized: ovarian cyst ruptured. Therefore, emphasize the necessity for heightened awareness for the possible correlation between SNRI therapies and AEs related to ovarian cysts.

**TABLE 4:** Identification of AE signals associated with reproductive toxicity of SNRIs in the male demographic.

Drug	Preferred terms	*N*	ROR (95% Two‐Sided CI)	PRR (95% Two‐Sided CI)	*χ* ^2^	IC (IC025)	EBGM (EBGM05)
Duloxetine	**Erectile dysfunction**	133	2.13 (1.79–2.52)	2.12 (1.95–2.29)	78.52	1.08 (−0.59)	2.12 (1.83)
	Sexual dysfunction^a^	130	6.22 (5.23–7.4)	6.21 (6.03–6.38)	557.08	2.61 (0.94)	6.11 (5.28)
	Ejaculation disorded	25	3.19 (2.15–4.73)	3.19 (2.8–3.58)	37.24	1.66 (0)	3.17 (2.28)
	Ejaculation failure^a^	19	3.64 (2.31–5.72)	3.64 (3.18–4.09)	35.9	1.85 (0.18)	3.61 (2.47)
	Priapism	16	1.81 (1.11–2.95)	1.81 (1.31–2.3)	5.73	0.85 (−0.82)	1.8 (1.19)
	Ejaculation delayed^a^	16	11.49 (6.97–18.91)	11.48 (10.98–11.98)	147.8	3.47 (1.8)	11.12 (7.32)
	Penis disorded	13	1.94 (1.12–3.34)	1.94 (1.39–2.48)	5.86	0.95 (−0.72)	1.93 (1.22)
	Pelvic pain	9	2.55 (1.32–4.91)	2.55 (1.89–3.2)	8.4	1.34 (−0.33)	2.54 (1.46)
	**Retrograde ejaculation** ^a^	8	4.48 (2.23–9.01)	4.48 (3.78–5.18)	21.34	2.15 (0.48)	4.43 (2.47)
	Testicular swelling	7	2.13 (1.01–4.48)	2.13 (1.39–2.87)	4.16	1.09 (−0.58)	2.12 (1.14)
	Prostatism^a^	6	15.09 (6.65–34.22)	15.09 (14.27–15.91)	75.36	3.85 (2.17)	14.45 (7.28)
	Male sexual dysfunction^a^	5	3.6 (1.49–8.7)	3.6 (2.72–4.49)	9.3	1.84 (0.17)	3.58 (1.71)
	Penile discharge^a^	4	5.46 (2.03–14.67)	5.46 (4.47–6.45)	14.32	2.43 (0.75)	5.38 (2.35)
	Genital burninng sensation^a^	3	3.71 (1.19–11.59)	3.71 (2.58–4.85)	5.88	1.88 (0.21)	3.68 (1.42)
	Pelvic haematoma^a^	3	8.71 (2.77–27.42)	8.71 ()7.56–9.86	19.93	3.09 (1.4)	8.5 (3.26)
Venlafaxine	**Erectile dysfunction** ^a^	250	4.72 (4.16–5.34)	4.7 (4.57–4.82)	718.93	2.22 (0.55)	4.65 (4.19)
	Sexual dysfunction^a^	132	7.39 (6.22–8.78)	7.38 (7.2–7.55)	713.62	2.86 (1.19)	7.25 (6.28)
	Ejaculation disorded^a^	29	4.34 (3.01–6.26)	4.34 (3.97–4.7)	73.65	2.1 (0.44)	4.3 (3.17)
	Priapism^a^	25	3.31 (2.24–4.91)	3.31 (2.92–3.71)	40.02	1.72 (0.05)	3.29 (2.37)
	Male sexual dysfunction^a^	18	15.65 (9.76–25.08)	15.64 (15.17–16.11)	236.8	3.91 (2.24)	15.05 (10.15)
	Ejaculation delayed^a^	16	13.44 (8.16–22.13)	13.44 (12.94–13.93)	177.77	3.7 (2.03)	13 (8.57)
	Ejaculation failure^a^	15	3.35 (2.02–5.57)	3.35 (2.84–3.86)	24.51	1.74 (0.07)	3.33 (2.18)
	Genital hypoaesthesia^a^	14	9.31 (5.48–15.83)	9.31 (8.78–9.84)	101.3	3.19 (1.52)	9.11 (5.84)
	Genital anesthesia^a^	11	65.26 (34.39–123.83)	65.24 (64.6–65.89)	592.43	5.8 (4.1)	55.69 (32.59)
	Penile size reduced^a^	8	3.77 (1.88–7.57)	3.77 (3.07–4.47)	16.12	1.9 (0.23)	3.74 (2.09)
	Spontaneous ejaculation^a^	8	74.75 (34.99–159.7)	74.74 (73.98–75.49)	484.99	5.96 (4.25)	62.45 (33.09)
	Testicular atrophy^a^	6	3.24 (1.45–7.23)	3.24 (2.43–4.04)	9.19	1.69 (0.02)	3.22 (1.64)
	**Retrograde ejaculation** ^a^	6	3.92 (1.75–8.76)	3.92 (3.12–4.72)	12.91	1.96 (0.29)	3.89 (1.98)
	Haematospermia^a^	6	3.9 (1.74–8.72)	3.9 (3.09–4.7)	12.8	1.95 (0.28)	3.87 (1.97)
	Neonatal testicular torsion^a^	5	1868.57 (218.29–15994.84)	1868.38 (1866.23–1870.52)	1555.31	8.29 (6.3)	312.23 (51.79)
	Painful ejaculation^a^	4	7.99 (2.97–21.52)	7.99 (7–8.98)	7.99	2.97 (1.29)	7.85 (3.43)
	Prostatism^a^	4	11.59 (4.28–31.35)	11.59 (10.59–12.58)	11.59	3.49 (1.81)	11.27 (4.9)
	Penile discomfort^a^	3	14.75 (4.65–46.76)	14.75 (13.6–15.9)	14.75	3.83 (2.13)	14.23 (5.42)

*Note:* Text in bold signifies that the signal is categorized as an important medical events (IMEs). IMEs are developed and updated by European Medicines Agency (EMA).

Abbreviations: CI, confidence interval; EBGM, empirical Bayesian geometric mean; IC, information component; IC025 and EBGM05, lower one‐sided for IC and EBGM, respectively; *N*, number of adverse event reported; PRR, proportional reporting ratio; ROR, reporting odds ratio; *χ* ^2^, chi‐squared.

^a^ Adhering to the four algorithms.

**TABLE 5:** Identification of AE signals associated with reproductive toxicity of SNRIs in the female demographic.

Drug	Preferred terms	*N*	ROR (95% two‐sided CI)	PRR (95% two‐sided CI)	*χ* ^2^	IC (IC025)	EBGM (EBGM05)
Duloxetine	**Sexual dysfunction** ^a^	99	8.05 (6.58–9.85)	8.05 (7.85–8.25)	582.6	2.95 (1.28)	7.72 (6.52)
	Female sexual dysfunction^a^	6	5.83 (2.58–13.17)	5.83 (5.02–6.65)	23.2	2.5 (0.82)	5.67 (2.87)
	Clitoral engorgement^a^	4	20.66 (7.3–58.41)	20.66 (19.62–21.69)	66.5	4.21 (2.48)	18.47 (7.74)
	Inadequate lubrication^a^	3	9.35 (2.92–29.93)	9.35 (8.19–10.52)	21.18	3.15 (1.45)	8.91 (3.36)
	Genital hypoaesthesia^a^	3	3.49 (1.11–10.95)	3.49 (2.35–4.63)	5.22	1.78 (0.1)	3.44 (1.32)
Venlafaxine	**Sexual dysfunction** ^a^	53	6.09 (4.63–7.99)	6.09 (5.81–6.36)	219.55	2.57 (0.91)	5.96 (4.74)
	Uterine disorded	21	2.56 (1.67–3.94)	2.56 (2.13–2.99)	19.79	1.35 (−0.32)	2.55 (1.78)
	Menopausal symptoms	15	2.08 (1.25–3.46)	2.08 (1.58–2.59)	8.38	1.05 (−0.61)	2.07 (1.36)
	Female sexual dysfunction^a^	13	19.03 (10.82–33.49)	19.03 (18.47–19.6)	205.69	4.15 (2.47)	17.7 (11.03)
	Female sexual arousal disorder^a^	5	17.05 (6.88–42.23)	17.04 (16.14–17.95)	70.48	4 (2.3)	15.98 (7.48)
	Menstrual discomfort^a^	5	14.91 (6.04–36.81)	14.91 (14.01–15.82)	61.09	3.82 (2.12)	14.1 (6.62)
	Genital anesthesia^a^	4	21.69 (7.79–60.38)	21.69 (20.67–22.72)	72.38	4.32 (2.6)	19.97 (8.48)
	Genital hypoaesthesia^a^	3	5.04 (1.61–15.82)	5.04 (3.9–6.18)	9.52	2.31 (0.63)	4.96 (1.9)

*Note:* Text in bold signifies that the signal is categorized as an important medical events (IMEs). IMEs are developed and updated by European Medicines Agency (EMA).

Abbreviations: CI, confidence interval; EBGM, empirical Bayesian geometric mean; IC, information component; IC025 and EBGM05, lower one‐sided for IC and EBGM, respectively; *N*, number of adverse event reported; PRR, proportional reporting ratio; ROR, reporting odds ratio; *χ* ^2^, chi‐squared.

^a^ Adhering to the four algorithms.

To investigate SNRI reproductive toxicity AEs, we discovered 27 PTs not listed in the labeling using Tables 3, 4, 5, Tables S5 and S6. In males, sexual dysfunction included priapism, retrograde ejaculation, spontaneous ejaculation, penile swelling, penile size reduced, penile discharge, and painful ejaculation. In females, sexual dysfunction involved clitoral engorgement, inadequate lubrication, and sexual arousal disorder. Testicular abnormalities such as swelling, atrophy, Neonatal testicular torsion, and hematospermia were noted, alongside prostate issues like prostatism. Ovarian conditions included hemorrhagic ovarian cysts and ovarian cyst ruptured, while genital sensory abnormalities comprised burning sensations, hypoesthesia, and anesthesia. Menstrual issues, such as menstrual discomfort, menometrorrhagia, oligomenorrhea, and vulvovaginal dryness, were also reported, as were menopausal symptoms and pelvic or genital injuries, including pelvic haematoma and penis disordered. These findings highlight a diverse range of reproductive system‐related AEs potentially associated with SNRI treatment.

### 3.3 TTO Of SNRI‐Related Erectile Dysfunction

**FIGURE 4:**
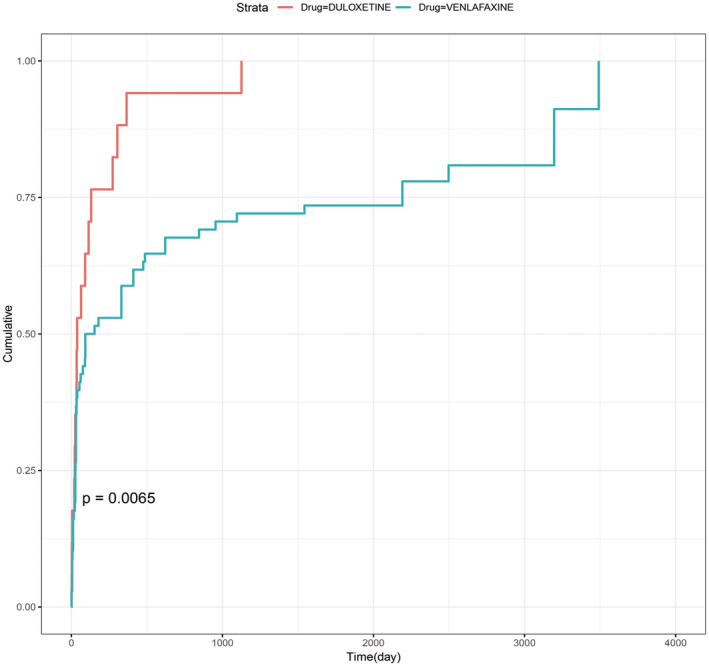
The cumulative incidence of erectile dysfunction in patients treated with duloxetine or venlafaxine. A comparison of the cumulative incidence of ED in individuals receiving venlafaxine and duloxetine revealed a statistically significant difference (log‐rank test, *p* = 0.0065).

We analyzed the onset times of ED related to venlafaxine and duloxetine. After excluding reports with inaccurate, missing, or unknown onset times, 17 duloxetine‐related and 68 venlafaxine‐related AEs provided onset times. For duloxetine, the median onset time was 38 days (IQR 20.5–202). For venlafaxine, it was 122.5 days (IQR 26–2191) (Table 6). Figure 4 demonstrates the cumulative incidence of ED associated with two SNRIs, showing a statistically significant difference (*p* = 0.0065). Within 30 days of starting duloxetine, 35.29% of patients experienced ED, and 76.46% within 180 days. For venlafaxine, 33.82% of patients had ED within 30 days, and 52.93% within 180 days. Notably, 11.76% of patients on duloxetine and 41.18% of those on venlafaxine might continue to experience ED 1 year after starting treatment.

**TABLE 6:** Time‐to‐onset analysis of SNRI‐related erectile dysfunction signals using the Weibull distribution test.

Drug	Time‐to‐onset (days)			Weibull distribution				Failure type
				Scale parameter		Shape parameter		
	*N*	Median (IQR)	Min–Max	*α*	95% CI	*β*	95% CI	
Duloxetine	17	38 (20.5–202)	2–1126	110	24.94–195.06	0.65	0.42–0.88	Early failure
Venlafaxine	68	122.5 (26–2191)	1–3491	500.55	245.67–755.42	0.49	0.40–0.59	Early failure

Abbreviations: CI, confidence interval; IQR, interquartile range; *N*, number of adverse event reported.

To assess whether the likelihood of SNRI‐related ED changes over time, we performed a WSP analysis (Table 6). Duloxetine and venlafaxine had shape parameters (*β*) of 0.65 (95% CI: 0.42–0.88) and 0.49 (95% CI: 0.40–0.58), respectively, which indicate early failure type and a declining incidence of ED over time.

## 4 Discussion

This pharmacovigilance study, utilizing real‐world FAERS data, offers updated evidence on the reproductive safety of venlafaxine and duloxetine. Reichenpfader et al. demonstrated that when evaluating male patients separately, the weighted average incidence rates for venlafaxine (12.30%) and duloxetine (10.80%) were higher, while the incidence rates for other SSRIs (such as escitalopram and fluoxetine) were below 10% [25]. Unfortunately, their meta‐analysis did not provide different results based on various stages of the sexual cycle. Although the association of SNRIs with male patients was increased, the impact on the overall population (including both males and females) was more limited. This might indicate that SNRIs play a specific role in male‐specific sexual dysfunctions, such as ED. Serretti and Chiesa [26] found that when assessing the impact on sexual dysfunction in both males and females, venlafaxine had the highest proportion of arousal dysfunction. Trinchieri et al. [14] observed a specific effect of SNRIs on ED when SSRIs and SNRIs were analyzed separately. Patients treated with SNRIs have a higher likelihood of developing ED compared to those receiving a placebo, whereas patients treated with SSRIs do not. Despite the recognition of antidepressants' association with ED, no pharmacovigilance analysis has specifically addressed SNRIs and ED [27]. This study found 261 cases of ED associated with venlafaxine and 135 with duloxetine, with venlafaxine showing stronger signal strength in pharmacovigilance algorithms (ROR, PRR, BCPNN, MGPS). We speculate that the mechanism of action of SNRIs may be connected to the association between ED and the drug. SNRIs raise 5‐HT and norepinephrine (NE) concentrations in the synaptic cleft, with 5‐HT generally believed to inhibit male sexual behavior through somatic, parasympathetic, and sympathetic efferent processes [28, 29]. Overconsumption of 5‐HT can lower arousal and sexual desire, which can impact erectile function. This is due to the fact that 5‐HT can block sexual response pathways by acting on specific central and peripheral 5‐HT receptors [30, 31, 32]. NE can worsen anxiety and stress, which can further impair erectile function [33]. The penile corpora cavernosa's blood engorgement is necessary for erectile function. The engorgement process may be impeded by the vasoconstrictive impact of NE, which can lower blood flow to the penile arteries and result in ED [34, 35]. Additionally, NE and 5‐HT may have an impact on the regulation of blood pressure, possibly leading to variations in blood pressure that harm endothelial function, hence diminishing penile blood flow and influencing erectile function [36, 37]. Furthermore, reports suggest that SNRIs may cause cardiovascular side effects, which can lead to anxiety and concern in patients, potentially triggering or worsening ED [38, 39, 40, 41].

In addition to ED, our analysis reveals that SNRIs significantly impact male reproductive AEs, including ejaculation disorders (e.g., delayed ejaculation, ejaculation failure, and retrograde ejaculation), genital sensory abnormalities, and testicular dysfunction. For females, SNRIs are strongly associated with sexual dysfunction, menstrual irregularities, and ovarian conditions such as hemorrhagic ovarian cysts. These findings corroborate existing knowledge of antidepressant‐related reproductive side effects [42, 43]. Notably, venlafaxine is typically more strongly linked to this reproductive harm than duloxetine. The longer half‐life of venlafaxine and its metabolite desvenlafaxine causes prolonged receptor interaction and hormonal abnormalities, which could account for the variations in reproductive toxicity between duloxetine and venlafaxine [44]. Additionally, testicular atrophy has been noted as a related adverse effect of venlafaxine, while clitoral erection and inadequate lubrication are associated with duloxetine use. However, these adverse reactions lack sufficient case reports and robust evidence, necessitating further investigation.

Descriptive studies suggest that females report AEs related to SNRI medications more frequently than males. However, when it comes to SNRI‐related reproductive toxicity, males report these adverse reactions more often than females, aligning with previous studies [45]. The mechanisms behind this gender difference are not fully understood, but several factors have been proposed. A plausible explanation is that the influence of estrogen renders women more vulnerable to symptoms like anxiety, depression, and eating disorders, thus increasing their susceptibility to the negative effects of SNRIs, thereby resulting in a greater likelihood of reporting AEs [46]. Additionally, some healthcare practitioners may perceive sexual dysfunction as predominantly a male concern, possibly leading to underreporting or greater acceptance of treatment‐induced sexual dysfunction in females [47, 48].

Age subgroup analysis revealed a high frequency of ED reports associated with both duloxetine and venlafaxine across all age groups, except for duloxetine in the < 45 years group, where no positive ED signal was detected. Time‐to‐onset analysis showed that within 180 days, ED occurred in 76.46% of duloxetine cases and 52.93% of venlafaxine cases. Both SNRIs exhibited early failure characteristics, suggesting that most patients experience ED within 6 months of treatment, with the risk gradually decreasing over time. Medical professionals should take into account the possible harmful effects on the reproduction of SNRIs and carefully weigh their effectiveness against safety, while also promptly addressing any negative side effects that may arise. These findings need to be confirmed through prospective studies and long‐term follow‐up.

The exact mechanisms by which SNRIs cause reproductive harm are unknown. However, SNRIs are thought to mostly affect the reproductive system by affecting serotonin and norepinephrine levels, disturbing neuroendocrine and neurotransmitter pathways essential to sexual function, hormonal balance, and fertility [49, 50, 51]. Inhibition of the serotonin transporter (SERT) raises serotonin levels, which bind to 5‐HT2A receptors in brain regions linked with sexual arousal, reducing dopamine signaling, which is necessary for sexual motivation and pleasure [52]. Sexual dysfunction, including decreased desire, anorgasmia, and delayed ejaculation, is frequent in both men and women due to dopaminergic pathway inhibition. On the other hand, increased norepinephrine levels can dysregulate the sympathetic nervous system (SNS), which controls sexual arousal and erectile performance [53]. Excess norepinephrine can overstimulate α1‐adrenergic receptors, causing vasoconstriction and decreased blood flow, potentially causing ED and ejaculatory problems. Serotonin modulates the production of gonadotropin‐releasing hormone (GnRH), which regulates the secretion of luteinizing hormone (LH) and follicle‐stimulating hormone (FSH) in the hypothalamic‐pituitary‐gonadal (HPG) axis [54]. These hormonal changes can disrupt female ovarian function, causing amenorrhea, oligomenorrhea, and anovulation, and male testicular function, causing hypogonadism and low testosterone levels, which further reduce fertility and sexual dysfunction. Serotonin and norepinephrine's impacts on neuroendocrine signaling, neurotransmitter control, and hormonal pathways cause reproductive toxicity, including sexual dysfunction, menstruation abnormalities, and fertility difficulties [55].

While our study offers significant insights into the reproductive toxicity linked to SNRIs, it is crucial to recognize its limitations. First, the inherent biases of the FAERS database due to its spontaneous reporting structure can lead to errors, underreporting, overreporting, inaccuracies, and delays, which may impact the precise assessment of drug‐related risks. Second, the FAERS database does not provide the total number of patients using SNRIs (denominator data), making it impossible to determine the exact incidence rate of reproductive system damage caused by these drugs. Third, the causal relationship between the reported AEs and drug exposure could not be confirmed [56]. This limitation is inherent in all pharmacovigilance studies, including those using the FAERS database. Fourth, as the FAERS database predominantly comprises reports from the U.S. and Europe, it may introduce racial biases and further clinical validation is needed to extend the conclusion to other races.

Notwithstanding these constraints, this work provides a thorough and methodical examination of potential hazards linked to reproductive toxicity generated by SNRIs. Through the utilization of big data and comprehensive analysis, we provide a valuable array of signals that can aid in the advancement of research and therapeutic practice in this particular subject.

## 5 Conclusion

We systematically examined the FAERS database to assess SNRI reproductive toxicity‐related AE risks. Venlafaxine has a higher risk of reproductive damage than duloxetine, especially in ED and ejaculatory disorders, and afflicted individuals are younger. These findings aid clinical decision‐making and drug monitoring. This study characterizes each SNRI's reproductive toxicity and population features. This information will assist clinicians understand each drug's reproductive toxicity and make better treatment judgments.

## Author Contributions

Jingkai Di and Ke Yang conceptualized and designed the study. Yujia Xi and Zhuocheng Bao conducted the data analysis and drafted the manuscript. Qiang Guo, Jingqi Wang, and Zhinan Jing were responsible for data collection, ensuring the integrity and accuracy of the data. All authors contributed to the interpretation of the data, revised the manuscript, and approved the final version.

## Conflicts of Interest

The authors declare no conflicts of interest.

## Supporting information


Table S1.

Table S2.

Table S3.

Table S4.

Table S5.

Table S6.

## Data Availability

The FAERS database, available at https://fis.fda.gov/extensions/FPD‐QDE‐FAERS/FPD‐QDE‐FAERS.html, is where the raw data used in this work were obtained.
