# Supplementary material for: Reproductive Toxicity Induced by Serotonin‐Norepinephrine Reuptake Inhibitors: A Pharmacovigilance Analysis From 2004 to 2023 Based on the FAERS Database
Source: CNS Neurosci Ther. 2024 Dec 13;30(12):e70176. doi: 10.1111/cns.70176 (PMC11638886; doi:10.1111/cns.70176)
Supplement: Supplementary file 1 — Table S1. [file CNS-30-e70176-s006.docx]

**Supplementary Table 1** Two-by-two contingency table

|  | Number of suspect adverse events | Number of other adverse events | Total |
| --- | --- | --- | --- |
| Suspect drug | a | b | a+b |
| Other drug | c | d | c+d |
| Total | a+c | b+d | a+b+c+d |

Abbreviations: a, number of reports containing both the suspect drug and the suspect adverse drug reaction; b, number of reports containing the suspect adverse drug reaction with other medications (except the drug of interest); c, number of reports containing the suspect drug with other adverse drug reactions (except the event of interest); d, number of reports containing other medications and other adverse drug reactions.
