# Supplementary material for: Reproductive Toxicity Induced by Serotonin‐Norepinephrine Reuptake Inhibitors: A Pharmacovigilance Analysis From 2004 to 2023 Based on the FAERS Database
Source: CNS Neurosci Ther. 2024 Dec 13;30(12):e70176. doi: 10.1111/cns.70176 (PMC11638886; doi:10.1111/cns.70176)
Supplement: Supplementary file 2 — Table S2. [file CNS-30-e70176-s004.docx]

**Supplementary Table 2** Four major algorithms used for signal detection

| Algorithms | Equation | Criteria |
| --- | --- | --- |
| ROR | ROR=ad/bc | lower limit of 95% CI>1, N≥3 |
|  | 95%CI=eln(ROR)±1.96(1/a+1/b+1/c+1/d)^0.5 |  |
| PRR | PRR=a(c+d)/c/(a+b) | PRR≥2, χ2≥4, N≥3 |
|  | χ2=[(ad-bc)^2](a+b+c+d)/[(a+b)(c+d)(a+c)(b+d)] |  |
| BCPNN | IC=log2a(a+b+c+d)/(a+c)/(a+b) | IC025>0, N≥3 |
|  | 95%CI=E(IC) ± 2V(IC)^0.5 |  |
|  | r=(a+b+c+d)^2/(a+b+1)/(a+c+1) |  |
|  | E(IC)=log2a(a+b+c+d)^2/(a+b+c+d+r)/(a+b)/(a+c) |  |
|  | V(IC)=1/ln2{(b+c+d+r-1)/(a+1)/(a+b+c+d+r+1)+(2+b+c+2d)/(a++b+1)/(a+b+c+d+r+3)} |  |
|  | IC025=E(IC)- 2V(IC)^0.5 |  |
| MGPS | EBGM=a(a+b+c+d)/(a+c)/(a+b) | EBGM05≥2,N≥3 |
|  | 95%CI=eln(EBGM)±1.96(1/a+1/b+1/c+1/d)^0.5 |  |
|  | EBGM05=eln(EBGM)-1.96(1/a+1/b+1/c+1/d)^0.5 |  |

Abbreviations: a, number of reports containing both the suspect drug and the suspect adverse drug reaction; b, number of reports containing the suspect adverse drug reaction with other medications (except the drug of interest); c, number of reports containing the suspect drug with other adverse drug reactions (except the event of interest); d, number of reports containing other medications and other adverse drug reactions. ROR, reporting odds ratio; CI, confidence interval; N, the number of co-occurrences; PRR, proportional reporting ratio; χ2, chi-squared; BCPNN, Bayesian confidence propagation neural network; IC, information component; IC025, the lower limit of the 95% CI of the IC; E (IC), the IC expectations; V (IC), the variance of IC; MGPS, multi-item gamma Poisson shrinker; EBGM, empirical Bayesian geometric mean; EBGM05, the lower limit of the 95% CI of EBGM.
