# Supplementary material for: Reproductive Toxicity Induced by Serotonin‐Norepinephrine Reuptake Inhibitors: A Pharmacovigilance Analysis From 2004 to 2023 Based on the FAERS Database
Source: CNS Neurosci Ther. 2024 Dec 13;30(12):e70176. doi: 10.1111/cns.70176 (PMC11638886; doi:10.1111/cns.70176)
Supplement: Supplementary file 3 — Table S3. [file CNS-30-e70176-s002.docx]

**Supplementary Table 3** ROR for all PTs within the SOC of reproductive system and breast disorders associated with duloxetine.

| PT | a | b | c | d | ROR | P-adjust |
| --- | --- | --- | --- | --- | --- | --- |
| SEXUAL DYSFUNCTION | 237 | 252559 | 9331 | 51897194 | 5.22 | ＜0.001 |
| ERECTILE DYSFUNCTION | 135 | 252661 | 21219 | 51885306 | 1.31 | 0.012 |
| VAGINAL HAEMORRHAGE | 61 | 252735 | 38206 | 51868319 | 0.33 | ＜0.001 |
| GALACTORRHOEA | 52 | 252744 | 6045 | 51900480 | 1.77 | ＜0.001 |
| HEAVY MENSTRUAL BLEEDING | 43 | 252753 | 23414 | 51883111 | 0.38 | ＜0.001 |
| AMENORRHOEA | 39 | 252757 | 14433 | 51892092 | 0.55 | 0.002 |
| MENSTRUAL DISORDER | 38 | 252758 | 6968 | 51899557 | 1.12 | 0.782 |
| BREAST PAIN | 37 | 252759 | 9451 | 51897074 | 0.8 | 0.438 |
| INTERMENSTRUAL BLEEDING | 29 | 252767 | 19079 | 51887446 | 0.31 | ＜0.001 |
| MENSTRUATION IRREGULAR | 26 | 252770 | 23699 | 51882826 | 0.23 | ＜0.001 |
| EJACULATION DISORDER | 26 | 252770 | 2641 | 51903884 | 2.02 | 0.003 |
| BREAST TENDERNESS | 25 | 252771 | 6881 | 51899644 | 0.75 | 0.384 |
| PRIAPISM | 23 | 252773 | 3032 | 51903493 | 1.56 | 0.160 |
| PELVIC PAIN | 23 | 252773 | 15352 | 51891173 | 0.31 | ＜0.001 |
| BENIGN PROSTATIC HYPERPLASIA | 20 | 252776 | 4595 | 51901930 | 0.89 | 0.875 |
| BREAST ENLARGEMENT | 19 | 252777 | 3264 | 51903261 | 1.2 | 0.764 |
| EJACULATION FAILURE | 19 | 252777 | 1750 | 51904775 | 2.23 | 0.004 |
| GYNAECOMASTIA | 18 | 252778 | 30004 | 51876521 | 0.12 | ＜0.001 |
| BREAST DISCHARGE | 18 | 252778 | 1535 | 51904990 | 2.41 | 0.002 |
| BREAST SWELLING | 17 | 252779 | 2393 | 51904132 | 1.46 | 0.367 |
| EJACULATION DELAYED | 16 | 252780 | 476 | 51906049 | 6.9 | ＜0.001 |
| OVARIAN CYST | 15 | 252781 | 9013 | 51897512 | 0.34 | ＜0.001 |
| VULVOVAGINAL DRYNESS | 13 | 252783 | 2737 | 51903788 | 0.98 | 1.000 |
| PENIS DISORDER | 13 | 252783 | 2248 | 51904277 | 1.19 | 0.849 |
| PROSTATIC DISORDER | 12 | 252784 | 4959 | 51901566 | 0.5 | 0.073 |
| MENSTRUATION DELAYED | 12 | 252784 | 9239 | 51897286 | 0.27 | ＜0.001 |
| ENDOMETRIOSIS | 12 | 252784 | 4092 | 51902433 | 0.6 | 0.282 |
| TESTICULAR PAIN | 11 | 252785 | 2649 | 51903876 | 0.85 | 0.875 |
| LACTATION DISORDER | 11 | 252785 | 426 | 51906099 | 5.3 | ＜0.001 |
| DYSMENORRHOEA | 10 | 252786 | 10936 | 51895589 | 0.19 | ＜0.001 |
| OLIGOMENORRHOEA | 10 | 252786 | 1950 | 51904575 | 1.05 | 1.000 |
| MENOPAUSAL SYMPTOMS | 10 | 252786 | 1855 | 51904670 | 1.11 | 1.000 |
| VAGINAL DISCHARGE | 9 | 252787 | 8875 | 51897650 | 0.21 | ＜0.001 |
| BREAST DISCOMFORT | 9 | 252787 | 902 | 51905623 | 2.05 | 0.167 |
| BREAST MASS | 8 | 252788 | 5802 | 51900723 | 0.28 | 0.002 |
| RETROGRADE EJACULATION | 8 | 252788 | 598 | 51905927 | 2.75 | 0.047 |
| PREMENSTRUAL SYNDROME | 8 | 252788 | 1189 | 51905336 | 1.38 | 0.724 |
| PROSTATOMEGALY | 8 | 252788 | 3341 | 51903184 | 0.49 | 0.185 |
| MENOMETRORRHAGIA | 8 | 252788 | 1567 | 51904958 | 1.05 | 1.000 |
| PROSTATITIS | 7 | 252789 | 2673 | 51903852 | 0.54 | 0.333 |
| POLYMENORRHOEA | 7 | 252789 | 2603 | 51903922 | 0.55 | 0.357 |
| TESTICULAR SWELLING | 7 | 252789 | 1085 | 51905440 | 1.32 | 0.821 |
| HAEMORRHAGIC OVARIAN CYST | 7 | 252789 | 506 | 51906019 | 2.84 | 0.058 |
| BREAST CYST | 6 | 252790 | 1584 | 51904941 | 0.78 | 0.872 |
| ERECTION INCREASED | 6 | 252790 | 4093 | 51902432 | 0.3 | 0.014 |
| GENITAL PAIN | 6 | 252790 | 1281 | 51905244 | 0.96 | 1.000 |
| FEMALE SEXUAL DYSFUNCTION | 6 | 252790 | 177 | 51906348 | 6.96 | 0.002 |
| PROSTATISM | 6 | 252790 | 131 | 51906394 | 9.4 | 0.001 |
| UTERINE HAEMORRHAGE | 6 | 252790 | 3728 | 51902797 | 0.33 | 0.029 |
| GENITAL HYPOAESTHESIA | 6 | 252790 | 755 | 51905770 | 1.63 | 0.424 |
| CYSTOCELE | 5 | 252791 | 1901 | 51904624 | 0.54 | 0.449 |
| GENITAL HAEMORRHAGE | 5 | 252791 | 11369 | 51895156 | 0.09 | ＜0.001 |
| CERVIX DISORDER | 5 | 252791 | 646 | 51905879 | 1.59 | 0.498 |
| VULVOVAGINAL PAIN | 5 | 252791 | 4188 | 51902337 | 0.25 | 0.006 |
| NIPPLE PAIN | 5 | 252791 | 1733 | 51904792 | 0.59 | 0.571 |
| UTERINE DISORDER | 5 | 252791 | 2088 | 51904437 | 0.49 | 0.356 |
| MALE SEXUAL DYSFUNCTION | 5 | 252791 | 471 | 51906054 | 2.18 | 0.264 |
| PELVIC HAEMATOMA | 5 | 252791 | 377 | 51906148 | 2.72 | 0.148 |
| PAINFUL ERECTION | 4 | 252792 | 638 | 51905887 | 1.29 | 0.787 |
| PEYRONIE'S DISEASE | 4 | 252792 | 757 | 51905768 | 1.08 | 0.946 |
| PENILE DISCHARGE | 4 | 252792 | 244 | 51906281 | 3.37 | 0.130 |
| GENITAL BURNING SENSATION | 4 | 252792 | 711 | 51905814 | 1.16 | 0.946 |
| VULVOVAGINAL PRURITUS | 4 | 252792 | 4230 | 51902295 | 0.19 | 0.003 |
| VAGINAL ODOUR | 4 | 252792 | 1120 | 51905405 | 0.73 | 0.875 |
| PENILE SWELLING | 4 | 252792 | 1123 | 51905402 | 0.73 | 0.875 |
| CLITORAL ENGORGEMENT | 4 | 252792 | 34 | 51906491 | 24.16 | ＜0.001 |
| PREMATURE MENOPAUSE | 3 | 252793 | 774 | 51905751 | 0.8 | 1.000 |
| DYSPAREUNIA | 3 | 252793 | 3739 | 51902786 | 0.16 | 0.004 |
| TESTICULAR DISORDER | 3 | 252793 | 894 | 51905631 | 0.69 | 0.946 |
| VULVOVAGINAL DISCOMFORT | 3 | 252793 | 3775 | 51902750 | 0.16 | 0.003 |
| VAGINAL DISORDER | 3 | 252793 | 853 | 51905672 | 0.72 | 0.946 |
| GENITAL PARAESTHESIA | 3 | 252793 | 235 | 51906290 | 2.62 | 0.308 |
| OVARIAN DISORDER | 3 | 252793 | 844 | 51905681 | 0.73 | 0.946 |
| INADEQUATE LUBRICATION | 3 | 252793 | 59 | 51906466 | 10.44 | 0.018 |
| PENILE SIZE REDUCED | 3 | 252793 | 838 | 51905687 | 0.74 | 0.946 |
| POSTMENOPAUSAL HAEMORRHAGE | 3 | 252793 | 2012 | 51904513 | 0.31 | 0.160 |
| OVARIAN CYST RUPTURED | 3 | 252793 | 1105 | 51905420 | 0.56 | 0.662 |
| UTERINE SPASM | 3 | 252793 | 1645 | 51904880 | 0.37 | 0.308 |
| BREAST ENGORGEMENT | 3 | 252793 | 272 | 51906253 | 2.26 | 0.357 |
| POLYCYSTIC OVARIES | 3 | 252793 | 1523 | 51905002 | 0.4 | 0.357 |
| ENDOMETRIAL HYPERTROPHY | 2 | 252794 | 385 | 51906140 | 1.07 | 0.886 |
| SPONTANEOUS EJACULATION | 2 | 252794 | 50 | 51906475 | 8.21 | 0.106 |
| RECTOCELE | 2 | 252794 | 557 | 51905968 | 0.74 | 1.000 |
| PAINFUL EJACULATION | 2 | 252794 | 196 | 51906329 | 2.1 | 0.498 |
| ATROPHIC VULVOVAGINITIS | 2 | 252794 | 621 | 51905904 | 0.66 | 0.946 |
| VAGINAL CYST | 2 | 252794 | 233 | 51906292 | 1.76 | 0.571 |
| INFERTILITY | 2 | 252794 | 1362 | 51905163 | 0.3 | 0.308 |
| FEMALE SEXUAL AROUSAL DISORDER | 2 | 252794 | 79 | 51906446 | 5.2 | 0.193 |
| MENOPAUSAL DISORDER | 2 | 252794 | 103 | 51906422 | 3.99 | 0.279 |
| NIPPLE DISORDER | 2 | 252794 | 570 | 51905955 | 0.72 | 1.000 |
| SEMEN DISCOLOURATION | 2 | 252794 | 213 | 51906312 | 1.93 | 0.521 |
| ABNORMAL UTERINE BLEEDING | 2 | 252794 | 2081 | 51904444 | 0.2 | 0.069 |
| REPRODUCTIVE TRACT DISORDER | 2 | 252794 | 362 | 51906163 | 1.13 | 0.875 |
| SCROTAL DISORDER | 2 | 252794 | 279 | 51906246 | 1.47 | 0.651 |
| VULVOVAGINAL BURNING SENSATION | 2 | 252794 | 5459 | 51901066 | 0.08 | ＜0.001 |
| UTERINE PROLAPSE | 2 | 252794 | 933 | 51905592 | 0.44 | 0.599 |
| ENDOMETRIAL HYPERPLASIA | 2 | 252794 | 718 | 51905807 | 0.57 | 0.821 |
| GENITAL DISCOMFORT | 2 | 252794 | 458 | 51906067 | 0.9 | 1.000 |
| PELVIC DISCOMFORT | 2 | 252794 | 787 | 51905738 | 0.52 | 0.821 |
| SCROTAL PAIN | 2 | 252794 | 480 | 51906045 | 0.86 | 1.000 |
| ORGANIC ERECTILE DYSFUNCTION | 2 | 252794 | 354 | 51906171 | 1.16 | 0.875 |
| SPONTANEOUS PENILE ERECTION | 2 | 252794 | 559 | 51905966 | 0.73 | 1.000 |
| INFERTILITY FEMALE | 2 | 252794 | 1157 | 51905368 | 0.35 | 0.418 |
| UTERINE CYST | 2 | 252794 | 502 | 51906023 | 0.82 | 1.000 |
| MAMMARY DUCT ECTASIA | 1 | 252795 | 39 | 51906486 | 5.26 | 0.400 |
| SCROTAL OEDEMA | 1 | 252795 | 636 | 51905889 | 0.32 | 0.640 |
| ASTHENOSPERMIA | 1 | 252795 | 59 | 51906466 | 3.48 | 0.498 |
| HAEMATOSPERMIA | 1 | 252795 | 616 | 51905909 | 0.33 | 0.640 |
| VAGINAL ULCERATION | 1 | 252795 | 361 | 51906164 | 0.57 | 1.000 |
| NOCTURNAL EMISSION | 1 | 252795 | 28 | 51906497 | 7.33 | 0.340 |
| LACTATION INSUFFICIENCY | 1 | 252795 | 793 | 51905732 | 0.26 | 0.429 |
| NIPPLE INFLAMMATION | 1 | 252795 | 47 | 51906478 | 4.37 | 0.438 |
| NIPPLE SWELLING | 1 | 252795 | 230 | 51906295 | 0.89 | 1.000 |
| PENILE PAIN | 1 | 252795 | 2839 | 51903686 | 0.07 | 0.005 |
| CERVICAL CYST | 1 | 252795 | 192 | 51906333 | 1.07 | 0.821 |
| BREAST CALCIFICATIONS | 1 | 252795 | 863 | 51905662 | 0.24 | 0.356 |
| MALE REPRODUCTIVE TRACT DISORDER | 1 | 252795 | 292 | 51906233 | 0.7 | 1.000 |
| TESTICULAR MICROLITHIASIS | 1 | 252795 | 24 | 51906501 | 8.56 | 0.311 |
| TESTICULAR OEDEMA | 1 | 252795 | 112 | 51906413 | 1.83 | 0.662 |
| VULVA CYST | 1 | 252795 | 85 | 51906440 | 2.42 | 0.599 |
| SPERMATORRHOEA | 1 | 252795 | 11 | 51906514 | 18.67 | 0.188 |
| HYPOMENORRHOEA | 1 | 252795 | 2238 | 51904287 | 0.09 | 0.022 |
| BREAST OEDEMA | 1 | 252795 | 164 | 51906361 | 1.25 | 0.782 |
| OVULATION DISORDER | 1 | 252795 | 97 | 51906428 | 2.12 | 0.640 |
| PROSTATIC ATROPHY | 1 | 252795 | 18 | 51906507 | 11.41 | 0.271 |
| BREAST ATROPHY | 1 | 252795 | 195 | 51906330 | 1.05 | 0.821 |
| BREAST INDURATION | 1 | 252795 | 164 | 51906361 | 1.25 | 0.782 |
| BREAST DISORDER FEMALE | 1 | 252795 | 237 | 51906288 | 0.87 | 1.000 |
| OVARIAN MASS | 1 | 252795 | 634 | 51905891 | 0.32 | 0.640 |
| ENDOMETRIAL THICKENING | 1 | 252795 | 412 | 51906113 | 0.5 | 0.900 |
| SCROTAL ERYTHEMA | 1 | 252795 | 140 | 51906385 | 1.47 | 0.742 |
| SCROTAL SWELLING | 1 | 252795 | 816 | 51905709 | 0.25 | 0.429 |
| GENITAL BLISTER | 1 | 252795 | 194 | 51906331 | 1.06 | 0.821 |
| PREPUCE REDUNDANT | 1 | 252795 | 31 | 51906494 | 6.62 | 0.356 |
| UTERINE HAEMATOMA | 1 | 252795 | 108 | 51906417 | 1.9 | 0.662 |
| PENILE BURNING SENSATION | 1 | 252795 | 244 | 51906281 | 0.84 | 1.000 |
| PELVIC ORGAN PROLAPSE | 1 | 252795 | 221 | 51906304 | 0.93 | 1.000 |
| VULVOVAGINAL DISORDER | 1 | 252795 | 53 | 51906472 | 3.87 | 0.470 |
| GENITAL ANAESTHESIA | 1 | 252795 | 124 | 51906401 | 1.66 | 0.704 |
| GENITAL ATROPHY | 1 | 252795 | 83 | 51906442 | 2.47 | 0.599 |
| VULVAL ECZEMA | 1 | 252795 | 7 | 51906518 | 29.33 | 0.145 |
| GENITAL SWELLING | 1 | 252795 | 537 | 51905988 | 0.38 | 0.779 |
| PREMENSTRUAL DYSPHORIC DISORDER | 1 | 252795 | 125 | 51906400 | 1.64 | 0.704 |
| ADNEXA UTERI CYST | 1 | 252795 | 133 | 51906392 | 1.54 | 0.724 |
| GENITAL LESION | 1 | 252795 | 360 | 51906165 | 0.57 | 1.000 |
| TESTICULAR RETRACTION | 1 | 252795 | 61 | 51906464 | 3.37 | 0.505 |
| EPIDIDYMAL DISORDER | 1 | 252795 | 20 | 51906505 | 10.27 | 0.282 |
| PRURITUS GENITAL | 1 | 252795 | 2197 | 51904328 | 0.09 | 0.024 |
| BREAST DISORDER MALE | 1 | 252795 | 109 | 51906416 | 1.88 | 0.662 |
| UTERINE ATROPHY | 1 | 252795 | 67 | 51906458 | 3.06 | 0.521 |
| PROSTATIC HAEMORRHAGE | 1 | 252795 | 283 | 51906242 | 0.73 | 1.000 |
| COLPOCELE | 1 | 252795 | 26 | 51906499 | 7.9 | 0.329 |
| BREAST FIBROSIS | 1 | 252795 | 112 | 51906413 | 1.83 | 0.662 |
| OVARIAN HAEMATOMA | 1 | 252795 | 16 | 51906509 | 12.83 | 0.253 |
| AZOOSPERMIA | 1 | 252795 | 364 | 51906161 | 0.56 | 1.000 |
| INFERTILITY MALE | 1 | 252795 | 410 | 51906115 | 0.5 | 1.000 |
| TESTICULAR ATROPHY | 1 | 252795 | 725 | 51905800 | 0.28 | 0.521 |
| OEDEMA GENITAL | 1 | 252795 | 582 | 51905943 | 0.35 | 0.782 |
| VULVOVAGINAL EXFOLIATION | 1 | 252795 | 88 | 51906437 | 2.33 | 0.609 |
| VULVOVAGINAL SWELLING | 1 | 252795 | 1894 | 51904631 | 0.11 | 0.049 |
| FIBROCYSTIC BREAST DISEASE | 1 | 252795 | 709 | 51905816 | 0.29 | 0.521 |
| VAGINAL LESION | 1 | 252795 | 273 | 51906252 | 0.75 | 1.000 |
| BREAST HAEMORRHAGE | 1 | 252795 | 287 | 51906238 | 0.72 | 1.000 |

Abbreviations: PT, preferred term; a, number of reports containing both the suspect drug and the suspect adverse drug reaction; b, number of reports containing the suspect adverse drug reaction with other medications (except the drug of interest); c, number of reports containing the suspect drug with other adverse drug reactions (except the event of interest); d, number of reports containing other medications and other adverse drug reactions.

P-adjust is the P-value after Fisher's exact test or Chi-square test, adjusted for False Discovery Rate (FDR)
