# Supplementary material for: Reproductive Toxicity Induced by Serotonin‐Norepinephrine Reuptake Inhibitors: A Pharmacovigilance Analysis From 2004 to 2023 Based on the FAERS Database
Source: CNS Neurosci Ther. 2024 Dec 13;30(12):e70176. doi: 10.1111/cns.70176 (PMC11638886; doi:10.1111/cns.70176)
Supplement: Supplementary file 4 — Table S4. [file CNS-30-e70176-s003.docx]

**Supplementary Table 4** ROR for all PTs within the SOC of reproductive system and breast disorders associated with venlafaxine.

| PT | a | b | c | d | ROR | P-adjust |
| --- | --- | --- | --- | --- | --- | --- |
| ERECTILE DYSFUNCTION | 261 | 183974 | 21093 | 51953993 | 3.49 | ＜0.001 |
| SEXUAL DYSFUNCTION | 204 | 184031 | 9364 | 51965722 | 6.15 | ＜0.001 |
| GALACTORRHOEA | 64 | 184171 | 6033 | 51969053 | 2.99 | ＜0.001 |
| AMENORRHOEA | 38 | 184197 | 14434 | 51960652 | 0.74 | 0.170 |
| HEAVY MENSTRUAL BLEEDING | 38 | 184197 | 23419 | 51951667 | 0.46 | ＜0.001 |
| VAGINAL HAEMORRHAGE | 36 | 184199 | 38231 | 51936855 | 0.27 | ＜0.001 |
| INTERMENSTRUAL BLEEDING | 31 | 184204 | 19077 | 51956009 | 0.46 | ＜0.001 |
| EJACULATION DISORDER | 29 | 184206 | 2638 | 51972448 | 3.1 | ＜0.001 |
| PRIAPISM | 26 | 184209 | 3029 | 51972057 | 2.42 | ＜0.001 |
| MENSTRUATION IRREGULAR | 24 | 184211 | 23701 | 51951385 | 0.29 | ＜0.001 |
| UTERINE DISORDER | 21 | 184214 | 2072 | 51973014 | 2.86 | ＜0.001 |
| MALE SEXUAL DYSFUNCTION | 19 | 184216 | 457 | 51974629 | 11.73 | ＜0.001 |
| EJACULATION FAILURE | 18 | 184217 | 1751 | 51973335 | 2.9 | ＜0.001 |
| EJACULATION DELAYED | 18 | 184217 | 474 | 51974612 | 10.71 | ＜0.001 |
| GENITAL HYPOAESTHESIA | 18 | 184217 | 743 | 51974343 | 6.84 | ＜0.001 |
| VULVOVAGINAL DRYNESS | 17 | 184218 | 2733 | 51972353 | 1.75 | 0.082 |
| MENSTRUAL DISORDER | 16 | 184219 | 6990 | 51968096 | 0.65 | 0.204 |
| PELVIC PAIN | 16 | 184219 | 15359 | 51959727 | 0.29 | ＜0.001 |
| MENOPAUSAL SYMPTOMS | 16 | 184219 | 1849 | 51973237 | 2.44 | 0.002 |
| BREAST PAIN | 15 | 184220 | 9473 | 51965613 | 0.45 | 0.008 |
| GENITAL ANAESTHESIA | 15 | 184220 | 110 | 51974976 | 38.47 | ＜0.001 |
| FEMALE SEXUAL DYSFUNCTION | 13 | 184222 | 170 | 51974916 | 21.57 | ＜0.001 |
| BREAST CYST | 12 | 184223 | 1578 | 51973508 | 2.15 | 0.040 |
| POSTMENOPAUSAL HAEMORRHAGE | 11 | 184224 | 2004 | 51973082 | 1.55 | 0.383 |
| GENITAL HAEMORRHAGE | 11 | 184224 | 11363 | 51963723 | 0.27 | ＜0.001 |
| GYNAECOMASTIA | 11 | 184224 | 30011 | 51945075 | 0.1 | ＜0.001 |
| BENIGN PROSTATIC HYPERPLASIA | 11 | 184224 | 4604 | 51970482 | 0.67 | 0.400 |
| LACTATION DISORDER | 11 | 184224 | 426 | 51974660 | 7.28 | ＜0.001 |
| BREAST DISCHARGE | 10 | 184225 | 1543 | 51973543 | 1.83 | 0.187 |
| UTERINE HAEMORRHAGE | 9 | 184226 | 3725 | 51971361 | 0.68 | 0.475 |
| VULVOVAGINAL DISCOMFORT | 9 | 184226 | 3769 | 51971317 | 0.67 | 0.459 |
| MENOMETRORRHAGIA | 9 | 184226 | 1566 | 51973520 | 1.62 | 0.388 |
| DYSMENORRHOEA | 9 | 184226 | 10937 | 51964149 | 0.23 | ＜0.001 |
| BREAST DISORDER | 8 | 184227 | 1796 | 51973290 | 1.26 | 0.773 |
| PENILE SIZE REDUCED | 8 | 184227 | 833 | 51974253 | 2.71 | 0.035 |
| OVARIAN CYST | 8 | 184227 | 9020 | 51966066 | 0.25 | ＜0.001 |
| SPONTANEOUS EJACULATION | 8 | 184227 | 44 | 51975042 | 51.3 | ＜0.001 |
| MENSTRUATION DELAYED | 8 | 184227 | 9243 | 51965843 | 0.24 | ＜0.001 |
| BREAST MASS | 8 | 184227 | 5802 | 51969284 | 0.39 | 0.026 |
| PROSTATIC DISORDER | 7 | 184228 | 4964 | 51970122 | 0.4 | 0.048 |
| BREAST ENLARGEMENT | 7 | 184228 | 3276 | 51971810 | 0.6 | 0.400 |
| TESTICULAR PAIN | 7 | 184228 | 2653 | 51972433 | 0.74 | 0.683 |
| PROSTATOMEGALY | 7 | 184228 | 3342 | 51971744 | 0.59 | 0.384 |
| PENIS DISORDER | 7 | 184228 | 2254 | 51972832 | 0.88 | 0.977 |
| GENITAL PAIN | 7 | 184228 | 1280 | 51973806 | 1.54 | 0.400 |
| RETROGRADE EJACULATION | 7 | 184228 | 599 | 51974487 | 3.3 | 0.022 |
| VAGINAL DISCHARGE | 6 | 184229 | 8878 | 51966208 | 0.19 | ＜0.001 |
| DYSPAREUNIA | 6 | 184229 | 3736 | 51971350 | 0.45 | 0.144 |
| TESTICULAR ATROPHY | 6 | 184229 | 720 | 51974366 | 2.35 | 0.118 |
| HAEMATOSPERMIA | 6 | 184229 | 611 | 51974475 | 2.77 | 0.068 |
| FEMALE SEXUAL AROUSAL DISORDER | 5 | 184230 | 76 | 51975010 | 18.56 | ＜0.001 |
| PENILE PAIN | 5 | 184230 | 2835 | 51972251 | 0.5 | 0.297 |
| NEONATAL TESTICULAR TORSION | 5 | 184230 | 2 | 51975084 | 705.3 | ＜0.001 |
| PROSTATISM | 5 | 184230 | 132 | 51974954 | 10.69 | 0.001 |
| MENSTRUAL DISCOMFORT | 5 | 184230 | 89 | 51974997 | 15.85 | ＜0.001 |
| NIPPLE PAIN | 4 | 184231 | 1734 | 51973352 | 0.65 | 0.675 |
| VULVOVAGINAL PAIN | 4 | 184231 | 4189 | 51970897 | 0.27 | 0.025 |
| BREAST DISCOMFORT | 4 | 184231 | 907 | 51974179 | 1.24 | 0.697 |
| PAINFUL ERECTION | 4 | 184231 | 638 | 51974448 | 1.77 | 0.462 |
| PAINFUL EJACULATION | 4 | 184231 | 194 | 51974892 | 5.82 | 0.020 |
| CYSTOCELE | 4 | 184231 | 1902 | 51973184 | 0.59 | 0.552 |
| ENDOMETRIOSIS | 4 | 184231 | 4100 | 51970986 | 0.28 | 0.028 |
| GENITAL RASH | 3 | 184232 | 1226 | 51973860 | 0.69 | 0.921 |
| RECTOCELE | 3 | 184232 | 556 | 51974530 | 1.52 | 0.621 |
| PREMENSTRUAL SYNDROME | 3 | 184232 | 1194 | 51973892 | 0.71 | 0.921 |
| INFERTILITY | 3 | 184232 | 1361 | 51973725 | 0.62 | 0.766 |
| INFERTILITY MALE | 3 | 184232 | 408 | 51974678 | 2.07 | 0.345 |
| POLYMENORRHOEA | 3 | 184232 | 2607 | 51972479 | 0.32 | 0.137 |
| FEMALE GENITAL TRACT FISTULA | 3 | 184232 | 1402 | 51973684 | 0.6 | 0.674 |
| VULVOVAGINAL PRURITUS | 3 | 184232 | 4231 | 51970855 | 0.2 | 0.012 |
| GENITAL PARAESTHESIA | 3 | 184232 | 235 | 51974851 | 3.6 | 0.128 |
| POLYCYSTIC OVARIES | 3 | 184232 | 1523 | 51973563 | 0.56 | 0.580 |
| OVARIAN CYST RUPTURED | 3 | 184232 | 1105 | 51973981 | 0.77 | 1.000 |
| PENILE DISCOMFORT | 3 | 184232 | 81 | 51975005 | 10.45 | 0.013 |
| ERECTION INCREASED | 3 | 184232 | 4096 | 51970990 | 0.21 | 0.014 |
| PROSTATITIS | 3 | 184232 | 2677 | 51972409 | 0.32 | 0.128 |
| PREMATURE MENOPAUSE | 3 | 184232 | 774 | 51974312 | 1.09 | 0.887 |
| VULVOVAGINAL BURNING SENSATION | 2 | 184233 | 5459 | 51969627 | 0.1 | 0.001 |
| MENOPAUSAL DISORDER | 2 | 184233 | 103 | 51974983 | 5.48 | 0.128 |
| SHORTENED CERVIX | 2 | 184233 | 277 | 51974809 | 2.04 | 0.417 |
| PELVIC HAEMATOMA | 2 | 184233 | 380 | 51974706 | 1.48 | 0.552 |
| PEYRONIE'S DISEASE | 2 | 184233 | 759 | 51974327 | 0.74 | 1.000 |
| CERVIX DISORDER | 2 | 184233 | 649 | 51974437 | 0.87 | 1.000 |
| BREAST ENGORGEMENT | 2 | 184233 | 273 | 51974813 | 2.07 | 0.415 |
| UTERINE POLYP | 2 | 184233 | 1490 | 51973596 | 0.38 | 0.400 |
| TESTICULAR OEDEMA | 2 | 184233 | 111 | 51974975 | 5.08 | 0.139 |
| VAGINAL DISORDER | 2 | 184233 | 854 | 51974232 | 0.66 | 0.900 |
| ATROPHIC VULVOVAGINITIS | 2 | 184233 | 621 | 51974465 | 0.91 | 1.000 |
| PENILE DISCHARGE | 2 | 184233 | 246 | 51974840 | 2.29 | 0.394 |
| OLIGOMENORRHOEA | 2 | 184233 | 1958 | 51973128 | 0.29 | 0.197 |
| HYPOMENORRHOEA | 2 | 184233 | 2237 | 51972849 | 0.25 | 0.128 |
| BREAST TENDERNESS | 2 | 184233 | 6904 | 51968182 | 0.08 | ＜0.001 |
| OEDEMA GENITAL | 2 | 184233 | 581 | 51974505 | 0.97 | 1.000 |
| FIBROCYSTIC BREAST DISEASE | 2 | 184233 | 708 | 51974378 | 0.8 | 1.000 |
| INADEQUATE LUBRICATION | 2 | 184233 | 60 | 51975026 | 9.4 | 0.060 |
| PELVIC ORGAN PROLAPSE | 1 | 184234 | 221 | 51974865 | 1.28 | 0.684 |
| VULVAL OEDEMA | 1 | 184234 | 375 | 51974711 | 0.75 | 1.000 |
| LACTATION INSUFFICIENCY | 1 | 184234 | 793 | 51974293 | 0.36 | 0.683 |
| BREAST DISORDER FEMALE | 1 | 184234 | 237 | 51974849 | 1.19 | 0.697 |
| GENITAL EROSION | 1 | 184234 | 250 | 51974836 | 1.13 | 0.714 |
| ORGANIC ERECTILE DYSFUNCTION | 1 | 184234 | 355 | 51974731 | 0.79 | 1.000 |
| PROSTATIC PAIN | 1 | 184234 | 354 | 51974732 | 0.8 | 1.000 |
| BREAST HAEMORRHAGE | 1 | 184234 | 287 | 51974799 | 0.98 | 1.000 |
| HAEMATOCOELE FEMALE | 1 | 184234 | 0 | 51975086 | NA | 0.013 |
| GENITAL DYSAESTHESIA | 1 | 184234 | 12 | 51975074 | 23.51 | 0.118 |
| VAGINAL PROLAPSE | 1 | 184234 | 369 | 51974717 | 0.76 | 1.000 |
| OVARIAN DISORDER | 1 | 184234 | 846 | 51974240 | 0.33 | 0.552 |
| PELVIC DISCOMFORT | 1 | 184234 | 788 | 51974298 | 0.36 | 0.683 |
| VULVOVAGINAL SWELLING | 1 | 184234 | 1894 | 51973192 | 0.15 | 0.118 |
| VAGINAL LESION | 1 | 184234 | 273 | 51974813 | 1.03 | 0.746 |
| GENITAL ULCERATION | 1 | 184234 | 543 | 51974543 | 0.52 | 1.000 |
| ECTROPION OF CERVIX | 1 | 184234 | 76 | 51975010 | 3.71 | 0.403 |
| PERINEAL ULCERATION | 1 | 184234 | 111 | 51974975 | 2.54 | 0.498 |
| VULVAL ULCERATION | 1 | 184234 | 237 | 51974849 | 1.19 | 0.697 |
| COITAL BLEEDING | 1 | 184234 | 1305 | 51973781 | 0.22 | 0.209 |
| SPONTANEOUS PENILE ERECTION | 1 | 184234 | 560 | 51974526 | 0.5 | 1.000 |
| TESTICULAR DISORDER | 1 | 184234 | 896 | 51974190 | 0.31 | 0.552 |
| ENDOMETRIAL HYPERPLASIA | 1 | 184234 | 719 | 51974367 | 0.39 | 0.683 |
| TESTICULAR TORSION | 1 | 184234 | 120 | 51974966 | 2.35 | 0.524 |
| OVULATION PAIN | 1 | 184234 | 172 | 51974914 | 1.64 | 0.621 |
| BREAST DYSPLASIA | 1 | 184234 | 42 | 51975044 | 6.72 | 0.280 |
| BREAST SWELLING | 1 | 184234 | 2409 | 51972677 | 0.12 | 0.048 |
| SCROTAL PAIN | 1 | 184234 | 481 | 51974605 | 0.59 | 1.000 |
| BREAST HAEMATOMA | 1 | 184234 | 208 | 51974878 | 1.36 | 0.683 |
| SPERMATOGENESIS ABNORMAL | 1 | 184234 | 82 | 51975004 | 3.44 | 0.415 |
| BREAST CALCIFICATIONS | 1 | 184234 | 863 | 51974223 | 0.33 | 0.552 |
| SCROTAL HAEMATOCOELE | 1 | 184234 | 79 | 51975007 | 3.57 | 0.412 |
| UTERINE PAIN | 1 | 184234 | 828 | 51974258 | 0.34 | 0.552 |
| GALACTOSTASIS | 1 | 184234 | 39 | 51975047 | 7.23 | 0.266 |
| GENITAL SWELLING | 1 | 184234 | 537 | 51974549 | 0.53 | 1.000 |
| ASPERMIA | 1 | 184234 | 34 | 51975052 | 8.3 | 0.238 |
| NIPPLE EXUDATE BLOODY | 1 | 184234 | 165 | 51974921 | 1.71 | 0.615 |
| PERINEAL FISTULA | 1 | 184234 | 85 | 51975001 | 3.32 | 0.418 |
| VAGINAL ODOUR | 1 | 184234 | 1123 | 51973963 | 0.25 | 0.382 |
| CERVICAL DYSPLASIA | 1 | 184234 | 1872 | 51973214 | 0.15 | 0.118 |
| REPRODUCTIVE TRACT DISORDER | 1 | 184234 | 363 | 51974723 | 0.78 | 1.000 |
| ABNORMAL UTERINE BLEEDING | 1 | 184234 | 2082 | 51973004 | 0.14 | 0.084 |

Abbreviations: PT, preferred term; a, number of reports containing both the suspect drug and the suspect adverse drug reaction; b, number of reports containing the suspect adverse drug reaction with other medications (except the drug of interest); c, number of reports containing the suspect drug with other adverse drug reactions (except the event of interest); d, number of reports containing other medications and other adverse drug reactions.

P-adjust is the P-value after Fisher's exact test or Chi-square test, adjusted for False Discovery Rate (FDR)
