# Supplementary material for: Reproductive Toxicity Induced by Serotonin‐Norepinephrine Reuptake Inhibitors: A Pharmacovigilance Analysis From 2004 to 2023 Based on the FAERS Database
Source: CNS Neurosci Ther. 2024 Dec 13;30(12):e70176. doi: 10.1111/cns.70176 (PMC11638886; doi:10.1111/cns.70176)
Supplement: Supplementary file 5 — Table S5. [file CNS-30-e70176-s005.docx]

**Supplementary Table 5** An age-based subgroup analysis was utilized for duloxetine in order to identify adverse events linked to reproductive toxicity that occurred at least three counts.

| **AGE** | **PT** | **N** | **ROR  (95% Two-Sided CI)** | **PRR  (95% Two-Sided CI)** | **χ2** | **IC(IC025)** | **EBGM (EBGM05)** | |  |
| --- | --- | --- | --- | --- | --- | --- | --- | --- | --- |
| **＜45** | Sexual dysfunction* | 61 | 4.56 ( 3.54 - 5.88 ) | 4.56 ( 4.3 - 4.81 ) | 165.71 | 2.16 ( 0.5 ) | | 4.48 ( 3.62 ) | |
|  | Ejaculation disorder* | 9 | 3.67 ( 1.9 - 7.1 ) | 3.67 ( 3.01 - 4.33 ) | 17.21 | 1.86 ( 0.19 ) | | 3.63 ( 2.09 ) | |
|  | Cervix disorder | 4 | 2.7 ( 1.01 - 7.25 ) | 2.7 ( 1.72 - 3.69 ) | 4.24 | 1.42 ( -0.25 ) | | 2.68 ( 1.17 ) | |
|  | Female sexual dysfunction* | 4 | 9.27 ( 3.4 - 25.23 ) | 9.27 ( 8.26 - 10.27 ) | 28.22 | 3.16 ( 1.46 ) | | 8.91 ( 3.85 ) | |
|  | Penile swelling* | 4 | 4.48 ( 1.66 - 12.07 ) | 4.48 ( 3.49 - 5.47 ) | 10.59 | 2.14 ( 0.46 ) | | 4.41 ( 1.92 ) | |
|  | Ejaculation delayed* | 3 | 3.7 ( 1.18 - 11.6 ) | 3.7 ( 2.56 - 4.84 ) | 5.82 | 1.87 ( 0.19 ) | | 3.66 ( 1.41 ) | |
|  | **Retrograde ejaculation*** | 3 | 3.91 ( 1.25 - 12.27 ) | 3.91 ( 2.77 - 5.06 ) | 6.39 | 1.95 ( 0.27 ) | | 3.86 ( 1.48 ) | |
| **45 – 59** | Sexual dysfunction* | 57 | 8.39 ( 6.43 - 10.94 ) | 8.38 ( 8.11 - 8.64 ) | 353.57 | 3.01 ( 1.34 ) | | 8.04 ( 6.44 ) | |
|  | **Erectile dysfunction** | 39 | 1.83 ( 1.33 - 2.5 ) | 1.82 ( 1.51 - 2.14 ) | 14.39 | 0.86 ( -0.81 ) | | 1.82 ( 1.39 ) | |
|  | Ejaculation disorder | 8 | 2.16 ( 1.08 - 4.34 ) | 2.16 ( 1.47 - 2.86 ) | 4.94 | 1.1 ( -0.57 ) | | 2.15 ( 1.2 ) | |
|  | Ejaculation failure* | 7 | 5.59 ( 2.63 - 11.87 ) | 5.59 ( 4.84 - 6.34 ) | 25.58 | 2.45 ( 0.77 ) | | 5.45 ( 2.9 ) | |
|  | Prostatism* | 5 | 35.3 ( 13.51 - 92.23 ) | 35.3 ( 34.34 - 36.26 ) | 138.87 | 4.89 ( 3.14 ) | | 29.58 ( 13.25 ) | |
|  | Menometrorrhagia* | 5 | 5.52 ( 2.26 - 13.44 ) | 5.52 ( 4.63 - 6.41 ) | 17.93 | 2.43 ( 0.75 ) | | 5.38 ( 2.55 ) | |
|  | Oligomenorrhoea* | 4 | 6.25 ( 2.31 - 16.94 ) | 6.25 ( 5.25 - 7.25 ) | 17.03 | 2.6 ( 0.91 ) | | 6.07 ( 2.63 ) | |
|  | **Haemorrhagic ovarian cyst*** | 4 | 19.61 ( 6.98 - 55.11 ) | 19.61 ( 18.58 - 20.64 ) | 63.59 | 4.15 ( 2.42 ) | | 17.75 ( 7.48 ) | |
|  | Ejaculation delayed* | 3 | 7.16 ( 2.26 - 22.7 ) | 7.16 ( 6 - 8.31 ) | 15.27 | 2.79 ( 1.09 ) | | 6.92 ( 2.63 ) | |
|  | Pelvic haematoma* | 3 | 12.92 ( 4 - 41.71 ) | 12.92 ( 11.74 - 14.09 ) | 30.73 | 3.6 ( 1.88 ) | | 12.1 ( 4.54 ) | |
| **＞59** | Sexual dysfunction* | 22 | 10.75 ( 7.04 - 16.44 ) | 10.75 (10.33 - 11.17) | 188.76 | 3.39 ( 1.72 ) | | 10.46 ( 7.33 ) | |
|  | **Erectile dysfunction** | 18 | 1.6 ( 1.01 - 2.55 ) | 1.6 (1.14 - 2.07) | 4.06 | 0.68 ( -0.99 ) | | 1.6 ( 1.09 ) | |
|  | Pelvic pain | 12 | 2.69 ( 1.52 - 4.75 ) | 2.69 (2.12 - 3.26) | 12.65 | 1.42 ( -0.25 ) | | 2.68 ( 1.66 ) | |
|  | Ejaculation disorder* | 4 | 6.06 ( 2.25 - 16.28 ) | 6.06 (5.07 - 7.05) | 16.6 | 2.58 ( 0.9 ) | | 5.97 ( 2.61 ) | |
|  | Ejaculation failure* | 4 | 4.61 ( 1.72 - 12.36 ) | 4.61 (3.62 - 5.59) | 11.15 | 2.19 ( 0.52 ) | | 4.56 ( 2 ) | |

Abbreviations: N, number of adverse event reported; ROR, reporting odds ratio; CI, confidence interval; PRR, proportional reporting ratio; χ^2^, chi-squared; IC, information component; EBGM, empirical Bayesian geometric mean; IC025 and EBGM05, lower one-sided for IC and EBGM, respectively.

Text in bold signifies that the signal is categorized as an important medical events (IMEs). IMEs are developed and updated by European Medicines Agency (EMA).

*Adhering to the four algorithms.
