# Supplementary material for: Reproductive Toxicity Induced by Serotonin‐Norepinephrine Reuptake Inhibitors: A Pharmacovigilance Analysis From 2004 to 2023 Based on the FAERS Database
Source: CNS Neurosci Ther. 2024 Dec 13;30(12):e70176. doi: 10.1111/cns.70176 (PMC11638886; doi:10.1111/cns.70176)
Supplement: Supplementary file 6 — Table S6. [file CNS-30-e70176-s001.docx]

**Supplementary Table 6** An age-based subgroup analysis was utilized for venlafaxine in order to identify adverse events linked to reproductive toxicity that occurred at least three counts.

| **AGE** | **PT** | **N** | **ROR  (95% Two-Sided CI)** | **PRR  (95% Two-Sided CI)** | **χ2** | **IC(IC025)** | **EBGM**  **(EBGM05)** |
| --- | --- | --- | --- | --- | --- | --- | --- |
| **＜45** | **Erectile dysfunction*** | 121 | 4.34 ( 3.62 - 5.2 ) | 4.33 ( 4.15 - 4.51 ) | 302.63 | 2.09 ( 0.42 ) | 4.25 ( 3.65 ) |
|  | Sexual dysfunction* | 88 | 5.54 ( 4.48 - 6.85 ) | 5.54 ( 5.32 - 5.74 ) | 316.35 | 2.43 ( 0.76 ) | 5.39 ( 4.51 ) |
|  | Genital hypoaesthesia* | 17 | 7.07 ( 4.35 - 11.48 ) | 7.07 ( 6.58 - 7.55 ) | 85.06 | 2.77 ( 1.1 ) | 6.83 ( 4.55 ) |
|  | Male sexual dysfunction* | 11 | 10.79 ( 5.87 - 19.85 ) | 10.79 ( 10.18 - 11.4 ) | 91.96 | 3.35 ( 1.67 ) | 10.21 ( 6.13 ) |
|  | Genital anaesthesia* | 11 | 20.31 ( 10.87 - 37.95 ) | 20.31 ( 19.68 - 20.93 ) | 180.58 | 4.19 ( 2.5 ) | 18.27 ( 10.83 ) |
|  | Vulvovaginal dryness | 10 | 2.53 ( 1.36 - 4.73 ) | 2.53 ( 1.91 - 3.16 ) | 9.14 | 1.33 ( -0.34 ) | 2.51 ( 1.49 ) |
|  | Ejaculation disorder | 8 | 2.71 ( 1.35 - 5.46 ) | 2.71 ( 2.02 - 3.41 ) | 8.53 | 1.43 ( -0.24 ) | 2.69 ( 1.5 ) |
|  | Female sexual arousal disorder* | 5 | 30.66 ( 11.84 - 79.42 ) | 30.66 ( 29.71 - 31.61 ) | 121.73 | 4.71 ( 2.97 ) | 26.17 ( 11.8 ) |
|  | Ejaculation delayed* | 5 | 5.2 ( 2.14 - 12.67 ) | 5.2 ( 4.31 - 6.09 ) | 16.48 | 2.34 ( 0.66 ) | 5.08 ( 2.41 ) |
|  | Ejaculation failure | 5 | 2.56 ( 1.06 - 6.18 ) | 2.56 ( 1.67 - 3.44 ) | 4.66 | 1.34 ( -0.33 ) | 2.53 ( 1.21 ) |
|  | Testicular atrophy | 4 | 3.12 ( 1.16 - 8.39 ) | 3.12 ( 2.13 - 4.11 ) | 5.67 | 1.62 ( -0.05 ) | 3.08 ( 1.35 ) |
|  | Female sexual dysfunction* | 4 | 7.72 ( 2.83 - 21.02 ) | 7.72 ( 6.72 - 8.72 ) | 22.38 | 2.89 ( 1.2 ) | 7.43 ( 3.21 ) |
|  | Menstrual discomfort* | 4 | 17.61 ( 6.29 - 49.29 ) | 17.61 ( 16.58 - 18.64 ) | 56.84 | 4.01 ( 2.28 ) | 16.07 ( 6.79 ) |
|  | **Retrograde ejaculation** | 3 | 3.26 ( 1.04 - 10.22 ) | 3.26 ( 2.12 - 4.4 ) | 4.61 | 1.69 ( 0 ) | 3.22 ( 1.24 ) |
|  | Penile discomfort* | 3 | 22.4 ( 6.72 - 74.6 ) | 22.4 ( 21.19 - 23.6 ) | 54.25 | 4.32 ( 2.56 ) | 19.93 ( 7.28 ) |
| **45 – 59** | **Erectile dysfunction*** | 71 | 4.02 ( 3.18 - 5.08 ) | 4.01 (3.78 - 4.25) | 157.65 | 1.98 ( 0.32 ) | 3.96 ( 3.25 ) |
|  | Sexual dysfunction* | 48 | 8.4 ( 6.29 - 11.2 ) | 8.39 (8.1 - 8.67) | 300.4 | 3.02 ( 1.35 ) | 8.1 ( 6.37 ) |
|  | Priapism* | 13 | 4.87 ( 2.81 - 8.45 ) | 4.87 (4.32 - 5.42) | 39.12 | 2.26 ( 0.59 ) | 4.79 ( 3.02 ) |
|  | Ejaculation disorder* | 9 | 2.92 ( 1.51 - 5.64 ) | 2.92 (2.26 - 3.58) | 11.19 | 1.53 ( -0.14 ) | 2.89 ( 1.67 ) |
|  | Ejaculation delayed | 9 | 27.99 ( 13.96 - 56.09 ) | 27.98 (27.29 - 28.68) | 206.78 | 4.63 ( 2.94 ) | 24.83 ( 13.88 ) |
|  | Menopausal symptoms | 8 | 2.47 ( 1.23 - 4.95 ) | 2.47 (1.77 - 3.16) | 6.89 | 1.29 ( -0.38 ) | 2.45 ( 1.37 ) |
|  | Female sexual dysfunction* | 7 | 77.9 ( 32.75 - 185.32 ) | 77.89 (77.02 - 78.75) | 388.26 | 5.84 ( 4.08 ) | 57.19 ( 27.69 ) |
|  | Uterine disorder | 6 | 2.79 ( 1.25 - 6.25 ) | 2.79 (1.99 - 3.6) | 6.82 | 1.47 ( -0.2 ) | 2.77 ( 1.41 ) |
|  | Vulvovaginal discomfort | 6 | 2.31 ( 1.04 - 5.18 ) | 2.31 (1.51 - 3.12) | 4.43 | 1.2 ( -0.47 ) | 2.3 ( 1.17 ) |
|  | Penile size reduced* | 5 | 18.55 ( 7.43 - 46.27 ) | 18.54 (17.63 - 19.46) | 76.3 | 4.1 ( 2.39 ) | 17.13 ( 7.97 ) |
|  | Spontaneous ejaculation* | 5 | 528.59 ( 102.55 - 2724.64 ) | 528.52 (526.88 - 530.16) | 752.18 | 7.25 ( 5.29 ) | 151.72 ( 38.47 ) |
|  | Painful ejaculation* | 4 | 31.32 ( 10.96 - 89.52 ) | 31.32 (30.27 - 32.37) | 102.26 | 4.78 ( 3.03 ) | 27.41 ( 11.38 ) |
|  | Prostatism* | 4 | 32.53 ( 11.35 - 93.21 ) | 32.52 (31.47 - 33.58) | 105.93 | 4.82 ( 3.08 ) | 28.32 ( 11.74 ) |
|  | **Ovarian cyst ruptured*** | 3 | 8.57 ( 2.7 - 27.19 ) | 8.57 (7.42 - 9.72) | 19.28 | 3.05 ( 1.35 ) | 8.28 ( 3.15 ) |
|  | Menometrorrhagia* | 3 | 3.92 ( 1.25 - 12.27 ) | 3.91 (2.77 - 5.06) | 6.39 | 1.95 ( 0.27 ) | 3.86 ( 1.49 ) |
| **＞59** | **Erectile dysfunction** | 20 | 2.17 ( 1.4 - 3.36 ) | 2.17 (1.73 - 2.6) | 12.48 | 1.11 ( -0.56 ) | 2.16 ( 1.49 ) |
|  | Sexual dysfunction* | 8 | 4.66 ( 2.32 - 9.36 ) | 4.66 (3.96 - 5.36) | 22.76 | 2.21 ( 0.54 ) | 4.62 ( 2.58 ) |
|  | Ejaculation failure* | 4 | 5.6 ( 2.09 - 15.02 ) | 5.6 (4.61 - 6.59) | 14.92 | 2.47 ( 0.8 ) | 5.54 ( 2.43 ) |
|  | Ejaculation disorder* | 3 | 5.5 ( 1.76 - 17.18 ) | 5.5 (4.36 - 6.64) | 10.9 | 2.44 ( 0.77 ) | 5.44 ( 2.1 ) |
|  | **Retrograde ejaculation*** | 3 | 10.17 ( 3.24 - 31.96 ) | 10.17 (9.02 - 11.31) | 24.22 | 3.32 ( 1.63 ) | 9.96 ( 3.82 ) |

Abbreviations: N, number of adverse event reported; ROR, reporting odds ratio; CI, confidence interval; PRR, proportional reporting ratio; χ^2^, chi-squared; IC, information component; EBGM, empirical Bayesian geometric mean; IC025 and EBGM05, lower one-sided for IC and EBGM, respectively.

Text in bold signifies that the signal is categorized as an important medical events (IMEs). IMEs are developed and updated by European Medicines Agency (EMA).

*Adhering to the four algorithms.
